# Inducible CRISPR/Cas systems in precision oncology: Current applications and future perspectives

**DOI:** 10.1002/ctm2.70720

**Published:** 2026-06-15

**Authors:** Ziliang Ding, Yukun Wei, Yong Han, Pengfei Gu

**Affiliations:** ^1^ Department of Thyroid Surgery Binzhou Medical University Hospital Binzhou Shandong PR China

**Keywords:** cancer therapy, CRISPR/Cas9, inducible gene editing, precision oncology, tumour microenvironment

## Abstract

**Background:**

Inducible CRISPR/Cas systems enable spatiotemporal control of genome editing in response to chemical, optical, biological, or physical stimuli. By restricting genome‐editing activity to defined conditions, these systems may reduce off‐target exposure and immune burden while improving tumor‐selective control, making them attractive tools for precision oncology.

**Main body:**

This review summarizes the molecular mechanisms, design principles, and current applications of inducible CRISPR/Cas systems in cancer research and therapy. These platforms are classified into chemically inducible, optogenetic, tumor microenvironment‐responsive, physically triggered, and logic‐gated systems. Regulatory strategies are discussed at multiple levels, including transcriptional control, post‐translational regulation, guide RNA engineering, and stimulus‐responsive delivery. Key applications include functional genomic screening, cancer modeling, therapeutic gene editing, immunotherapy enhancement, and combinatorial treatment strategies. We also examine current delivery approaches, including viral vectors, lipid nanoparticles, stimulus‐responsive nanocarriers, and biomimetic platforms.

**Conclusion:**

Inducible CRISPR/Cas systems represent a promising platform for next‐generation precision cancer therapy. However, substantial optimization and rigorous preclinical validation remain necessary to address challenges related to leaky expression, induction efficiency, tissue penetration, immunogenicity, and long‐term safety before clinical translation can be realized.

**Key points:**

- Inducible CRISPR/Cas systems enable conditional genome editing in precision oncology.
- Chemical, optical, TME‐responsive, physical, and logic‐gated systems offer distinct control features.
- Delivery, leakiness, immunogenicity, and safety remain key translational barriers.
- Ex vivo immune‐cell engineering and locoregional delivery may offer nearer‐term clinical routes.

## Graphical abstract

Inducible CRISPR/Cas systems enable spatiotemporal genome editing in precision oncology. Chemical, optical, physical, and microenvironmental stimuli provide distinct control strategies. Conditional activation may reduce off‐target exposure and basal nuclease activity. This review summarizes mechanisms, delivery barriers, and oncologic applications.

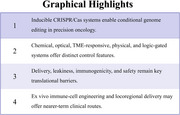

## 1 INTRODUCTION

Cancer remains one of the leading causes of morbidity and mortality worldwide, with an estimated 19.3 million new cases and nearly 10 million cancer‐related deaths reported annually.^1^ Despite remarkable advances in conventional therapeutic modalities—including surgery, chemotherapy, radiotherapy, and immunotherapy—the intrinsic heterogeneity, adaptive resistance, and metastatic potential of malignant tumours continue to pose formidable challenges to effective clinical management.^2^ Precision oncology aims to tailor treatment to the molecular and genetic features of individual tumours and has become an important direction in cancer treatment. A key requirement for this goal is the development of genome‐engineering tools that can identify, test, and modulate cancer‐associated genes with high specificity and efficiency.

The clustered regularly interspaced short palindromic repeats (CRISPR)/CRISPR‐associated protein (Cas) system, originally discovered as an adaptive immune mechanism in prokaryotes, has revolutionized the field of genome editing since its initial adaptation for mammalian genome engineering.^3^, ^4^, ^5^ The most widely employed CRISPR/Cas9 system, derived from Streptococcus pyogenes (SpCas9), utilizes a single guide RNA (sgRNA) to direct the Cas9 endonuclease to a complementary genomic target, where it introduces site‐specific double‐strand breaks (DSBs) that are subsequently repaired through non‐homologous end joining (NHEJ) or homology‐directed repair (HDR) pathways.^6^ Beyond the canonical Cas9 nuclease, the CRISPR toolkit has been substantially expanded to include catalytically impaired variants such as dead Cas9 (dCas9) for transcriptional regulation (CRISPRi/CRISPRa), base editors (BEs) for precise nucleotide conversions, prime editors (PEs) for versatile search‐and‐replace editing, and RNA‐targeting Cas13 systems.^7^, ^8^, ^9^ These diverse platforms have been extensively harnessed in oncology research for functional genomic screening, cancer modelling, biomarker discovery, and the development of novel therapeutic strategies.^10^, ^11^

However, the constitutive expression of CRISPR/Cas components raises several critical concerns that impede their translational application in cancer therapy. First, persistent and unregulated Cas nuclease activity substantially increases the risk of off‐target mutagenesis at unintended genomic loci, which may result in genotoxicity, chromosomal rearrangements, or even oncogenic transformation.^12^, ^13^ Second, prolonged exposure to bacterial‐derived Cas proteins can elicit adaptive immune responses in the host, potentially leading to the elimination of edited cells or adverse immunological reactions that compromise therapeutic efficacy.^14^, ^15^ Third, constitutive editing lacks the spatiotemporal precision required for preferentially targeting tumour cells while reducing damage to normal tissues—a prerequisite for limiting systemic toxicity in clinical settings.^16^ Together, these limitations highlight the need for controllable CRISPR/Cas platforms that can be activated or deactivated in a conditional, tunable, and reversible manner.

To address these challenges, a growing body of research has focused on the development of inducible CRISPR/Cas systems, in which the genome‐editing activity is placed under the control of exogenous stimuli. These stimuli include, but are not limited to, small‐molecule chemical inducers (e.g., doxycycline, trimethoprim, tamoxifen, rapamycin), light irradiation (optogenetic approaches), endogenous tumour‐specific signals (e.g., hypoxia, tumour‐specific promoters, microRNA profiles), and physical stimuli such as ultrasound and heat.^17^, ^18^, ^19^, ^20^ The strategies for achieving inducible control can be broadly categorized into transcriptional regulation, post‐translational regulation, sgRNA‐level control, and delivery‐level control. In transcriptional regulation, inducible promoters or synthetic gene circuits govern the expression of Cas proteins and/or sgRNAs. Post‐translational regulation employs chemically or optically induced protein dimerization, intein‐mediated protein splicing, or allosteric conformational switches to modulate Cas protein activity. sgRNA‐level control involves aptamer‐based, ligand‐responsive, or antisense‐mediated regulation of guide RNA availability and function. Delivery‐level control uses stimulus‐responsive nanocarriers or engineered viral vectors to enable conditional release of CRISPR components at target sites.^16^, ^21^ Each of these strategies offers distinct advantages in terms of temporal resolution, reversibility, multiplexability, and compatibility with in vivo applications, and several have demonstrated promising results in preclinical cancer models.

The combination of inducible CRISPR/Cas technology and precision oncology creates useful opportunities across several areas of cancer research and therapy. In the context of functional genomics, inducible systems enable the dissection of temporal gene dependencies during tumour initiation, progression, and metastasis, providing insights that are unattainable with constitutive knockout approaches.^22^ For cancer modelling, conditional CRISPR platforms allow the generation of sophisticated animal models that faithfully recapitulate the sequential acquisition of driver mutations observed in human tumorigenesis.^23^, ^24^ In therapeutic applications, stimulus‐responsive CRISPR systems offer the potential for tumour‐selective gene disruption, programmable activation of prodrug‐converting enzymes, conditional modulation of immune checkpoint pathways, and synergistic integration with existing treatment modalities such as photodynamic therapy (PDT) and photothermal therapy (PTT).^25^, ^26^ Moreover, logic‐gated and multi‐input inducible circuits provide a framework for developing “smart” gene therapies that can sense and respond to the complex tumour microenvironment (TME).^27^, ^28^

Despite these exciting developments, the clinical translation of inducible CRISPR/Cas systems in oncology faces several unresolved challenges, including the optimization of induction efficiency and dynamic range, the minimization of leaky expression in the uninduced state, the achievement of sufficient tissue penetration for physical stimuli such as light and ultrasound, the scalability of delivery vehicles for systemic administration, and the long‐term safety profiling of these multicomponent systems in vivo.^29^ Therefore, a critical evaluation of current progress is needed to guide future research and support the translation of these technologies toward clinical use.

**FIGURE 1:**
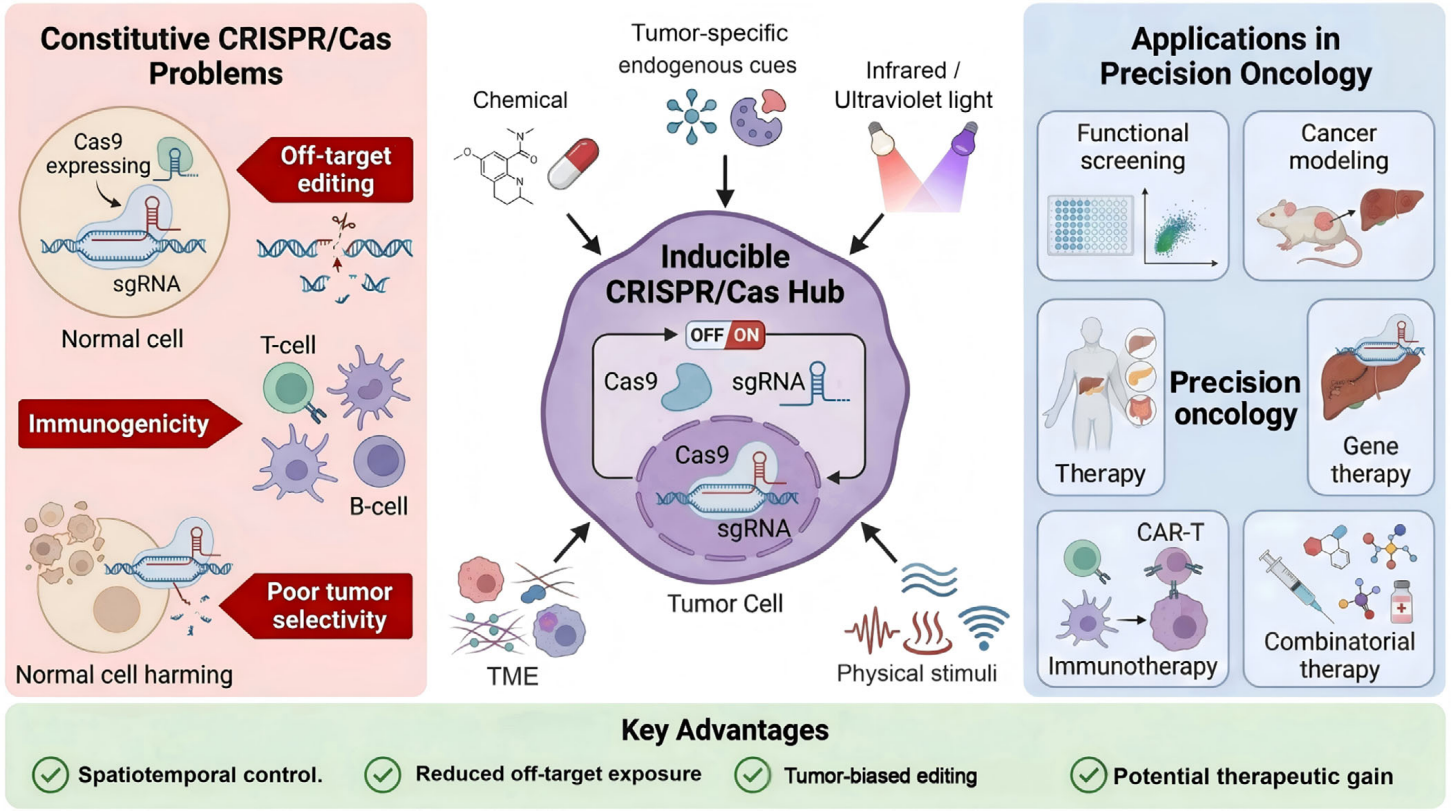
Overview of inducible CRISPR/Cas systems in precision oncology. Constitutively active CRISPR/Cas platforms may be limited by persistent nuclease activity, off‐target editing, immunogenicity, and insufficient tumour bias, which together increase the risk of damage to normal tissues. Inducible CRISPR/Cas systems are designed to address these limitations by enabling conditional activation of Cas proteins and/or sgRNAs in response to chemical inducers, light, tumour‐specific endogenous signals, tumour microenvironment cues, or other physical stimuli. This spatiotemporal control may reduce unintended nuclease exposure and improve the therapeutic window. Such systems have broad applications in functional genomic screening, cancer modelling, gene therapy, immunotherapy, and combinatorial treatment. Overall, inducible CRISPR/Cas strategies provide a controllable framework for precision genome engineering in oncology. This figure is a conceptual schematic based on the literature cited in the corresponding section and does not contain newly analyzed public datasets.

In this review, we provide a comprehensive and up‐to‐date overview of inducible CRISPR/Cas systems and their applications in precision oncology. We begin by summarizing the fundamental mechanisms and molecular architectures of different inducible strategies, including chemically inducible, optogenetically controlled, TME‐responsive, and physically triggered systems. We then discuss the current applications of these platforms across the cancer research continuum, encompassing functional genomic screening, cancer modelling, gene therapy, immunotherapy enhancement, and combinatorial therapeutic approaches. Finally, we critically examine the existing bottlenecks and propose future perspectives for the rational design, optimization, and clinical translation of next‐generation inducible CRISPR/Cas platforms in the fight against cancer. By linking recent advances in genome engineering with key clinical needs in oncology, this review aims to outline a practical roadmap for developing safer and more precise CRISPR‐based cancer therapeutics (Figure 1).

## 2 MECHANISMS AND CLASSIFICATION OF INDUCIBLE CRISPR/CAS SYSTEMS

### 2.1 Public data sources and data citation

This review did not generate new experimental data or perform an independent re‐analysis of public sequencing datasets. Publicly available information discussed in this manuscript was obtained from the original publications, public databases, and previously reported datasets cited in the corresponding sections, tables, and figure legends. When public resources were mentioned, the database name, accession number or web link, data type, sample information, and quality‐control criteria were described according to the original source whenever available. For figures and tables adapted from published or public resources, the corresponding references are cited in the figure legends and table captions. No individual‐level patient data were downloaded or reprocessed in this review.

Inducible CRISPR/Cas systems are developed mainly to provide spatiotemporal control over genome‐editing activity, thereby potentially reducing off‐target exposure, limiting immune burden, and improving tumour‐biased gene manipulation. Over the past decade, researchers have developed several inducible strategies that regulate one or more components of the CRISPR machinery through distinct molecular mechanisms. In this section, we systematically classify and discuss the principal categories of inducible CRISPR/Cas systems based on their triggering stimuli and regulatory mechanisms.

Conceptually, inducible CRISPR/Cas design can be understood at three related but different levels: inducible activity, inducible release, and tumour targeting. Inducible activity refers to direct regulation of Cas proteins or sgRNAs, such as transcriptional switches, split‐Cas reconstitution, destabilizing domains, or ligand‐responsive guide RNAs. Inducible release refers to stimulus‐triggered liberation of CRISPR cargo from delivery vehicles, such as pH‐, redox‐, enzyme‐, light‐, or ultrasound‐responsive nanocarriers. Tumour targeting refers to preferential accumulation or expression in malignant tissues through ligands, tumour‐specific promoters, membrane‐coated carriers, or cell‐based vehicles, but does not necessarily imply conditional activation of editing activity. Distinguishing these layers is important for evaluating specificity, safety, and translational feasibility.

To help readers compare these platforms more directly, Table S1 summarizes their ON/OFF control, leakiness, response kinetics, reversibility, in vivo compatibility, and translational feasibility.

### 2.2 Chemically inducible systems

Chemically inducible CRISPR/Cas systems represent the most extensively developed and widely adopted class of controllable genome‐editing platforms. These systems leverage small‐molecule compounds to modulate the expression, stability, localization, or catalytic activity of CRISPR components through a variety of molecular mechanisms (Table 1).

**TABLE 1:** Key features of inducible CRISPR/Cas platforms in precision oncology.

Induction type	Representative strategies	Control level	Kinetics/in vivo feasibility	Key advantages	Key limitations
Chemical	Tet‐ON/cumate switches; Split‐Cas9 CID; Inteins; destabilizing domains; ligand‐responsive sgRNAs	Transcriptional, Post‐translational, sgRNA	Minutes–hours; systemic but PK‐dependent	Mature; dose‐tunable; reversible	Leakiness; non‐target inducer exposure; Repeated dosing
Light	PaCas9/paCpf1; CRY2‐CIB1/magnet; Caged sgRNAs; NIR‐UCNP activation	Post‐translational, transcriptional, sgRNA, delivery	Seconds–minutes; high precision but limited depth	Excellent spatial control; minimal systemic inducer	Poor deep‐tissue penetration; phototoxicity; hardware/UCNP complexity
TME‐responsive	Tumour‐specific promoters; hypoxia/HIF circuits; miRNA switches; pH/GSH/ROS/enzyme‐triggered release	Transcriptional, translational, inhibitory, delivery	Hours–days; depends on TME gradients	Autonomous tumour‐biased activation; no external device	Tumour heterogeneity; weak output; inter‐patient variability
Physical	Ultrasound, FUS/heat, magnetic, or X‐ray‐triggered systems	Transcriptional, post‐translational, delivery	Minutes–hours; Better deep‐tissue potential	Remote non‐invasive triggering; compatible with local therapy	Device dependence; uneven energy deposition; safety calibration
Logic‐gated	AND‐gates integrating promoters, miRNAs, Acrs, or multiple TME cues	Circuit‐level	Slower; depends on input modules	Higher specificity; reduced false activation	Large payload; difficult delivery and optimization

*Note*: The information summarized in this table was extracted from published studies cited in the corresponding section. No independent public dataset re‐analysis was performed.

Abbreviations: Acr, anti‐CRISPR protein; CID, chemically induced dimerization; CRISPR, clustered regularly interspaced short palindromic repeats; FUS, focused ultrasound; GSH, glutathione; NIR, near‐infrared; PK, pharmacokinetics; ROS, reactive oxygen species; sgRNA, single‐guide RNA; TME, tumour microenvironment; UCNP, upconversion nanoparticle.

#### 2.2.1 Transcriptional induction

One of the most straightforward approaches to chemical induction involves placing the expression of Cas9 or sgRNA under the control of drug‐responsive promoters. The tetracycline (Tet)‐inducible system, comprising the reverse tetracycline‐controlled transactivator (rtTA) and the Tet‐responsive element (TRE), is perhaps the most widely used transcriptional platform for CRISPR regulation. In this system, the addition of doxycycline (Dox) induces conformational changes in rtTA that enable its binding to TRE, thereby activating transcription of the downstream Cas9 gene.^22^, ^30^ Dow and colleagues demonstrated that Dox‐inducible Cas9 expression in transgenic mice enabled efficient and reversible gene editing in vivo, providing a powerful platform for temporal genetic studies in cancer biology.^22^ Similarly, the cumate‐inducible system, based on the CymR repressor and the cumate operator sequence, has been adapted for conditional CRISPR/Cas9 expression in mammalian cells.^31^ These transcriptional induction systems offer the advantage of simplicity and compatibility with established genetic tools, but their temporal resolution is inherently limited by the kinetics of transcription, translation, and protein accumulation, typically requiring several hours to achieve maximal editing activity.

#### 2.2.2 Post‐translational chemical regulation

To achieve more rapid and precise control, several groups have developed post‐translational strategies in which Cas9 protein activity is directly modulated by small molecules.

Split‐Cas9 with chemically induced dimerization (CID): Zetsche et al.^32^ demonstrated that Cas9 can be split into two inactive fragments (N‐Cas9 and C‐Cas9) that are individually fused to rapamycin‐responsive dimerization domains (FKBP and FRB). In the absence of rapamycin, the two fragments remain separated and catalytically inactive; upon rapamycin administration, FKBP–FRB heterodimerization reconstitutes a functional Cas9 nuclease. This strategy has been further refined using alternative CID pairs such as the abscisic acid (ABA)‐responsive ABI/PYL1 system, providing orthogonal induction modalities.^33^

Intein‐mediated protein splicing: Intein‐based approaches involve the insertion of ligand‐dependent inteins into the Cas9 coding sequence, rendering the protein non‐functional until the appropriate small molecule triggers intein self‐splicing and reconstitution of full‐length Cas9. Davis et al.^17^ pioneered this concept by incorporating a 4‐hydroxytamoxifen (4‐HT)‐responsive intein into SpCas9, achieving dose‐dependent and temporally controlled editing with significantly reduced off‐target activity compared to constitutive Cas9. More recently, intein engineering and split‐intein strategies have been used to improve the inducibility, specificity, and portability of conditional Cas9 activity beyond the original 4‐HT design, providing alternative routes to tighten background activity and expand stimulus responsiveness.^34^

Destabilizing domain (DD)‐based regulation: The fusion of engineered destabilizing domains to Cas9 targets the protein for rapid proteasomal degradation in the absence of a stabilizing ligand. Upon addition of the cognate small molecule (e.g., Shield‐1 for the FKBP12‐derived DD, or trimethoprim (TMP) for the *E. coli* dihydrofolate reductase (ecDHFR)‐based DD), the DD undergoes conformational stabilization, preventing Cas9 degradation and enabling editing activity.^19^, ^35^ This strategy is particularly attractive for applications requiring reversible control, as removal of the stabilizing ligand leads to rapid Cas9 depletion.

Allosteric small‐molecule switches: Maji et al.^36^ developed an allosterically regulated Cas9 in which a small‐molecule‐dependent allosteric effector domain was engineered into the REC lobe of Cas9, such that ligand binding induces a conformational change that activates DNA cleavage. Similarly, anti‐CRISPR proteins (Acrs) have been adapted for chemically regulated inhibition: fusion of Acrs to degron domains enables ligand‐dependent degradation of the inhibitor, thereby activating CRISPR function.^37^, ^38^

#### 2.2.3 Chemical regulation at the sgRNA level

Chemical control can also be exerted at the guide RNA level. Ligand‐responsive aptamers incorporated into the sgRNA scaffold can modulate its structure and function in response to small molecules such as theophylline or tetracycline.^39^, ^40^ In the “off” state, the aptamer sequesters critical sgRNA regions required for Cas9 binding or target recognition; ligand binding to the aptamer releases these structural constraints, restoring sgRNA functionality. Kundert et al.^41^ systematically engineered ligand‐responsive sgRNAs with tunable dynamic ranges, demonstrating their utility for dose‐dependent gene regulation.

**FIGURE 2:**
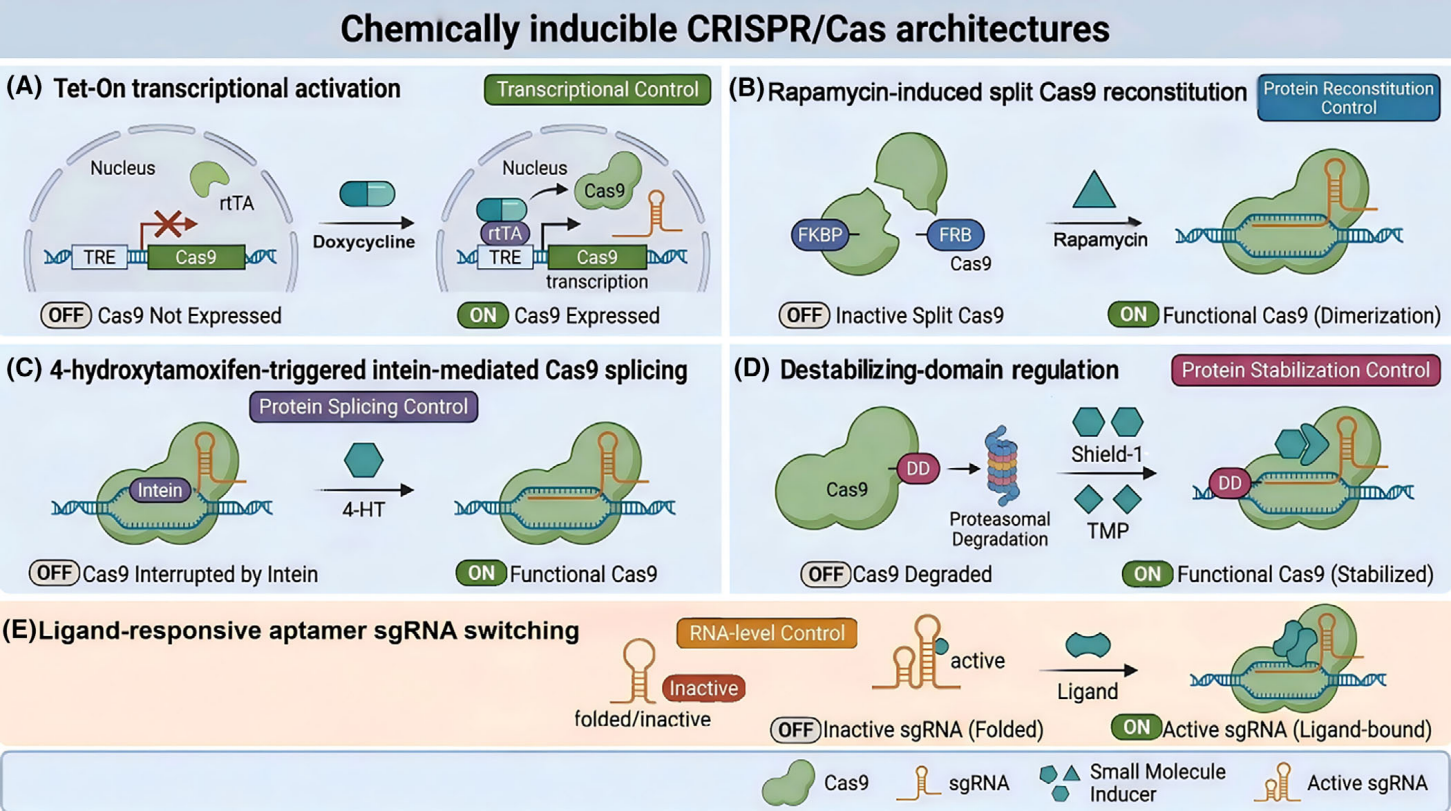
Representative architectures of chemically inducible CRISPR/Cas systems. Chemically inducible CRISPR/Cas platforms regulate genome‐editing activity through several molecular mechanisms. (A) Tet‐On transcriptional activation enables doxycycline‐dependent Cas9 expression. (B) Rapamycin‐induced dimerization reconstitutes split Cas9 and restores nuclease activity. (C) 4‐Hydroxytamoxifen triggers intein‐mediated protein splicing to generate functional Cas9. (D) Destabilizing‐domain regulation controls Cas9 abundance through ligand‐dependent protein stabilization. (E) Ligand‐responsive aptamer‐engineered sgRNAs enable RNA‐level switching by converting inactive folded guides into ligand‐bound active conformations. Together, these strategies illustrate how small molecules can provide tunable control over CRISPR/Cas activity at the transcriptional, post‐translational, and RNA levels. This figure is a conceptual schematic based on the literature cited in the corresponding section and does not contain newly analyzed public datasets.

Additionally, chemically modified sgRNAs with photolabile protecting groups (“caged” sgRNAs) or chemically cleavable modifications have been developed, although these approaches overlap with light‐inducible strategies and will be discussed in subsequent sections (Figure 2).^42^

### 2.3 Optogenetically controlled (light‐inducible) systems

Light‐inducible CRISPR/Cas systems exploit photosensitive protein domains or photolabile chemical groups to achieve rapid, reversible, and spatially defined genome editing. Light offers several unique advantages as an inducing stimulus: it is non‐invasive, can be delivered with high spatial precision (down to subcellular resolution), and enables near‐instantaneous switching kinetics.

#### 2.3.1 Photoactivatable split‐Cas9 systems

Nihongaki et al.^18^ pioneered the photoactivatable CRISPR‐Cas9 (paCas9) system by fusing split Cas9 fragments to the blue light‐responsive positive Magnet (pMag) and negative Magnet (nMag) photodimers derived from Neurospora crassa Vivid protein. Blue light illumination (488 nm) induces pMag–nMag heterodimerization and Cas9 reconstitution, enabling light‐dependent genome editing in human cells. The same group subsequently developed an improved version, paCas9‐2, with enhanced efficiency and reduced background activity.^43^ An alternative approach utilized the CRY2–CIB1 blue light‐inducible dimerization pair from Arabidopsis thaliana for split‐Cas9 reconstitution.^44^

#### 2.3.2 Light‐inducible transcriptional systems

Beyond direct Cas9 activation, light‐inducible transcription systems have been used to control the expression of CRISPR components. The light‐activated (LA)‐CRISPR system employs a split Cre recombinase fused to blue light‐responsive dimerization domains; upon illumination, Cre‐mediated recombination removes a transcriptional stop cassette flanked by loxP sites, permitting Cas9 expression from an upstream constitutive promoter.^44^ Similarly, the EL222 blue light‐responsive transcription factor has been adapted to drive light‐dependent Cas9 expression.^45^

#### 2.3.3 Caged sgRNAs and photocleavable modifications

An elegant chemical biology approach involves the introduction of photolabile protecting groups (“cages”) on critical nucleotides within the sgRNA, thereby blocking Cas9 loading or target hybridization in the dark. Upon UV or near‐visible light irradiation, the photolabile groups are cleaved, restoring sgRNA activity. Jain et al.^42^ demonstrated that caging as few as three nucleotides in the seed region of the sgRNA was sufficient to abolish editing activity in the dark, with full restoration upon brief UV exposure. Similarly, Hemphill et al.^46^ developed photocaged anti‐CRISPR oligonucleotides that inhibit sgRNA function in the dark and release it upon light exposure.

#### 2.3.4 Near‐infrared and upconversion nanoparticle‐mediated systems

Near‐infrared (NIR) light‐activated upconversion nanoparticle (UCNP) systems enable spatiotemporally precise control of CRISPR/Cas activity in deep tissues, overcoming limitations of conventional delivery. UCNPs convert tissue‐penetrating NIR light into localized UV/visible emissions to trigger photocleavable linkers or activate photosensitizers, facilitating endosomal escape and on‐demand release of CRISPR components. For instance, UCNP‐based nanomachines have been engineered for NIR‐controlled co‐delivery of CRISPR machinery and photosensitizers to enhance gene‐editing efficacy while enabling real‐time imaging guidance.^47^ Similarly, NIR‐upconversion theranostic platforms tailored for CRISPR‐Cas9/DNAzyme systems support precise tumour‐targeted gene regulation with minimized off‐target effects.^48^ Furthermore, UCNP‐integrated biosensors leverage CRISPR‐Cas12a for dual‐mode upconversion luminescence detection of nucleic acid biomarkers, enhancing diagnostic sensitivity in oncology applications.^49^

Despite these advantages, a representative problematic design is the use of UCNP‐assisted light‐inducible CRISPR activation for deep tumours. Although UCNPs can convert tissue‐penetrating NIR light into UV/visible emission and thereby activate photocaged or photosensitive CRISPR modules, as demonstrated in NIR‐responsive CRISPR/Cas9 nanocarrier systems, this design introduces several translational liabilities, including nanoparticle retention, uncertain long‐term clearance, local heating or phototoxicity, heterogeneous intratumoral distribution, and difficulty in standardizing the delivered optical dose.^50^ Thus, UCNP‐assisted systems are conceptually powerful for spatial control but remain less mature than chemically inducible or transient non‐viral delivery platforms for clinical oncology.

### 2.4 Biologically inducible systems

#### 2.4.1 Tumour‐specific promoter‐driven systems

Tissue‐ and tumour‐specific promoters represent a straightforward transcriptional approach to restrict CRISPR activity to cancer cells. Commonly used cancer‐specific promoters include the human telomerase reverse transcriptase (hTERT) promoter, the survivin promoter, the alpha‐fetoprotein (AFP) promoter for hepatocellular carcinoma, and the prostate‐specific antigen (PSA) promoter for prostate cancer.^27^ Liang et al.^51^ demonstrated that driving Cas9 expression from the hTERT promoter restricted gene editing to telomerase‐positive cancer cells while sparing normal cells. However, the relatively weak transcriptional activity of many tumour‐specific promoters, compared with constitutive promoters such as CMV or EF1α, remains a significant challenge that may necessitate the use of transcriptional amplification strategies.^27^

#### 2.4.2 Hypoxia‐responsive systems

Hypoxia, a hallmark of solid tumours, contributes to therapy resistance, metastasis, and immune evasion.^52^, ^53^ Hypoxia‐responsive CRISPR/Cas systems exploit hypoxia‐inducible factors (HIFs), particularly HIF‐1α—a master transcriptional regulator of cellular adaptation to low oxygen—to restrict genome‐editing activity or cargo release to hypoxic tumour regions. Current strategies include HIF‐responsive regulatory circuits, hypoxia‐activated nanoplatforms, tumour‐homing carriers delivering CRISPR/Cas9 against hypoxia‐associated targets, and oxygen‐generating or redox‐responsive systems combined with CRISPR‐mediated pathway modulation.^51^, ^53^, ^54^ These approaches may modulate hypoxia‐adaptive metabolic programs and immunosuppressive signalling, including the PD‐1/PD‐L1 axis, in specific experimental settings.^51^, ^53^ Overall, hypoxia‐responsive platforms convert a key tumour microenvironmental feature into a therapeutic trigger, potentially improving tumour selectivity while supporting combination with chemo‐, immuno‐, and phototherapeutic strategies.^52^, ^54^ Future work should focus on improving induction efficiency, delivery kinetics, and robustness across heterogeneous hypoxic niches.

#### 2.4.3 MicroRNA‐responsive systems

Differential microRNA (miRNA) expression profiles between cancer cells and normal cells can be exploited to construct miRNA‐responsive CRISPR circuits. Hirosawa et al. engineered miRNA‐responsive Cas9 mRNAs containing miRNA target sites in the 5′‐ or 3′‐untranslated regions (UTRs)^55^; in cells expressing the cognate miRNA, Cas9 translation is repressed, while in cells lacking the miRNA (e.g., cancer cells with downregulated tumour‐suppressive miRNAs), Cas9 is expressed. Conversely, Acr can be placed under miRNA‐responsive control, where cancer‐specific miRNAs degrade the anti‐CRISPR mRNA and de‐repress CRISPR activity. Hoffmann et al.^56^ demonstrated a cell‐type‐specific CRISPR system controlled by endogenous miR‐21, which is overexpressed in many cancers, achieving selective gene editing in miR‐21‐high tumour cells.

#### 2.4.4 Redox and pH‐responsive systems

The TME is characterized by elevated levels of reactive oxygen species (ROS), glutathione (GSH), and acidic extracellular pH (6.5–6.8) compared to normal tissues.^57^ These biochemical differences have been exploited for stimulus‐responsive delivery of CRISPR components. Liu et al. developed GSH‐responsive nanoparticles that release Cas9/sgRNA ribonucleoproteins (RNPs) selectively in the reducing intracellular environment of tumour cells.^58^ Similarly, pH‐sensitive lipid nanoparticles (LNPs) that undergo structural disassembly in the acidic TME or endosomal compartments have been engineered for tumour‐selective CRISPR delivery.^59^

Dynamic metabolic reprogramming represents another clinically relevant feature of the TME that may be exploited by inducible CRISPR/Cas systems. In bladder cancer, altered lipid metabolism, including fatty acid transport, fatty acid metabolism‐related molecular subtypes, and TERC‐driven fatty acid remodelling, has been associated with tumour progression, prognosis, immune microenvironment features, and cisplatin resistance.^60^, ^61^, ^62^ Because lipid metabolic pathways are also essential for normal tissue homeostasis, constitutive CRISPR‐mediated disruption of these axes may increase toxicity in non‐malignant cells. TME‐responsive or physically triggered inducible CRISPR platforms may therefore provide a safer strategy by restricting metabolic gene editing to specific tumour regions or therapeutic windows.

### 2.5 Physically triggered systems

Physical stimuli, including ultrasound, magnetic fields, and temperature, offer unique advantages for deep‐tissue induction of CRISPR activity with minimal chemical intervention.

#### 2.5.1 Ultrasound‐responsive systems

Ultrasound‐responsive systems offer a non‐invasive, spatiotemporally precise platform for inducible CRISPR/Cas applications. These systems leverage ultrasound's deep tissue penetration and stimuli‐responsive nanocarriers to enable on‐demand release of genetic payloads or activation of gene circuits.^63^, ^64^ Recent advances in ultrasound‐triggered transgene circuits provide a foundation for spatiotemporally regulated CRISPR/Cas activation.^65^ Integration with TME cues or sonodynamic therapy may further enhance specificity and therapeutic synergy.^66^ However, clinical translation requires refinement in safety, scalability, stimulus calibration, and delivery efficiency.^67^

#### 2.5.2 Thermal control systems

Thermal control systems enable spatiotemporally precise activation of CRISPR/Cas tools in oncology through non‐invasive external stimuli. Focused ultrasound (FUS) or NIR light induces localized mild hyperthermia (≈42°C), triggering Cas9‐mediated editing of apoptosis‐resistance genes (e.g., *HSP70*, *BAG3*) within tumours while sparing healthy tissue.^68^ This strategy disrupts tumour cell survival mechanisms and remodels the immunosuppressive microenvironment to enhance adoptive T‐cell infiltration and efficacy. Magnetothermal approaches similarly activate CRISPR‐Cas9 for targeted *HSP70* knockout, achieving synergistic pro‐apoptotic effects.^69^ Engineered thermal gene switches leveraging heat‐shock promoters (e.g., *HSP70*) and optimized FUS platforms provide reversible, tunable control with real‐time temperature monitoring.^70^

#### 2.5.3 Magnetic field‐responsive systems

**FIGURE 3:**
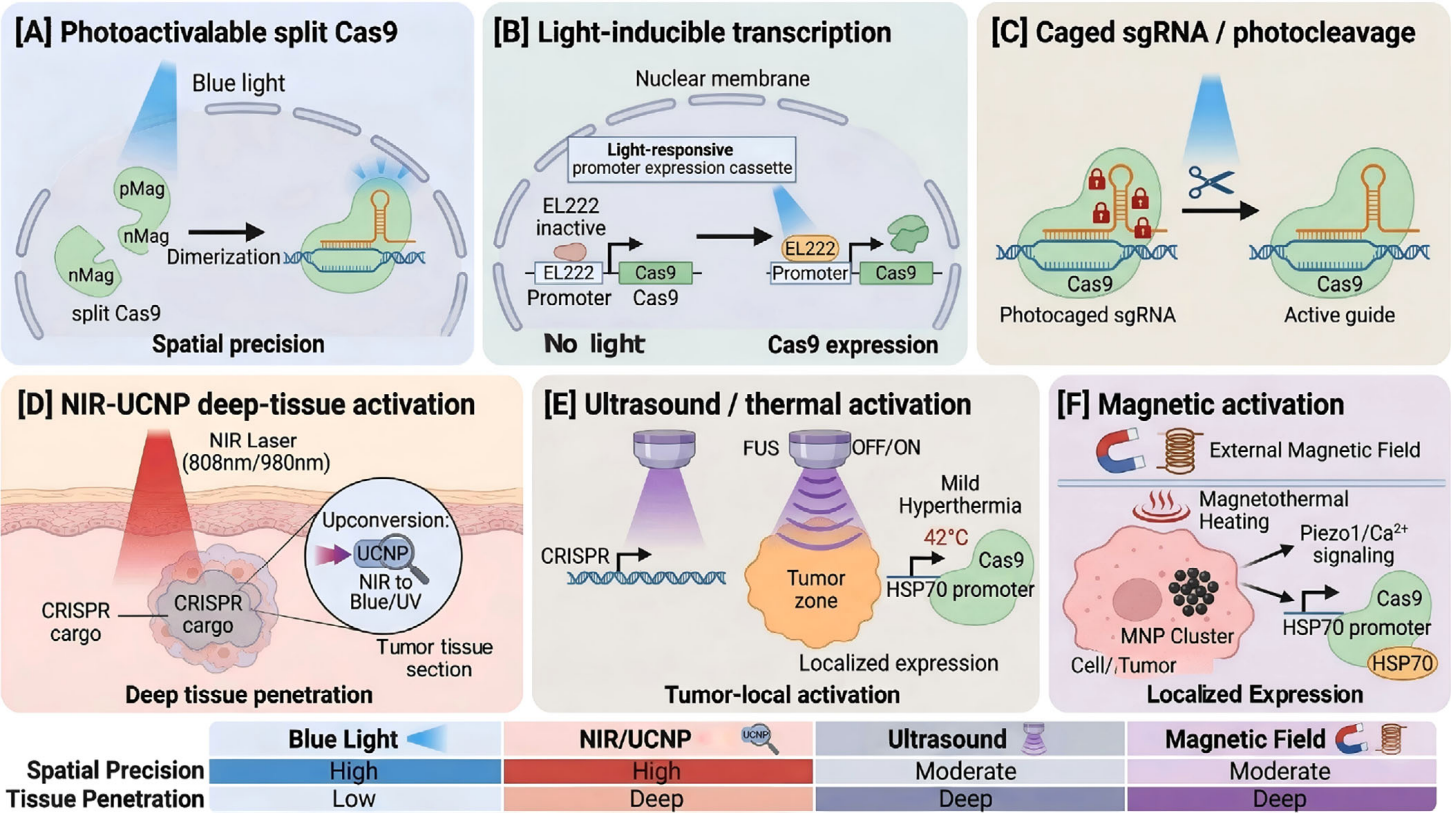
Light‐ and physical‐stimuli‐responsive CRISPR/Cas platforms for precision oncology. Optical and physical cues provide external control over CRISPR/Cas activity with different levels of spatial precision and tissue penetration. (A) Blue light induces dimerization of photoactivatable split Cas9 for localized genome editing. (B) Light‐responsive transcription factors such as EL222 enable optical regulation of Cas9 expression. (C) Photocaged sgRNAs are activated by light‐triggered cleavage. (D) Near‐infrared light combined with upconversion nanoparticles permits deeper tissue activation by converting NIR signals into shorter‐wavelength light. (E) FUS or mild hyperthermia activates heat‐responsive promoters to support tumour‐localized CRISPR expression. (F) magnetic‐field‐triggered magnetothermal signalling can also enables localized Cas9 activation. These platforms highlight the trade‐off between activation precision and tissue penetration, device dependence and potential safety constraints in externally controlled genome editing. This figure is a conceptual schematic based on the literature cited in the corresponding section and does not contain newly analyzed public datasets.

Magnetic field‐responsive CRISPR/Cas systems may support spatiotemporally controlled genome editing in oncology by leveraging external magnetic stimuli to activate editing machinery preferentially within tumour regions. Magnetothermal approaches utilize alternating magnetic fields to induce mild hyperthermia (∼42°C), triggering dual CRISPR‐Cas9 editing to disrupt apoptosis‐resistance genes (e.g., HSP70) while synergistically sensitizing tumour cells to thermal apoptosis.^69^ Magnetomechanical strategies employ magnetic nanostructures to activate mechanosensitive channels (e.g., Piezo1), initiating Ca^2^^+^‐dependent expression of Cas9 for remote, non‐invasive gene regulation.^71^ Additionally, magnetic nanoparticle‐assisted platforms (e.g., MAGE, magnetoplexes) enhance delivery efficiency, biocompatibility, and on‐target accuracy of CRISPR components to tumour sites.^72^, ^73^ These systems may reduce off‐target exposure and collateral damage to healthy tissues, which are important concerns for translating CRISPR‐based therapies to clinical oncology (Figure 3).^74^

### 2.6 Multi‐input logic‐gated systems

To enhance the specificity and sophistication of inducible CRISPR platforms, researchers have begun constructing multi‐input logic gates that integrate two or more conditional signals. Nissim et al.^27^ developed a multi‐layered genetic circuit combining tissue‐specific promoters, miRNA inputs, and CRISPR effectors to create AND‐gate logic, where CRISPR activity is activated only when all input conditions are simultaneously met. Such logic‐gated circuits may improve tumour selectivity by requiring the coincidence of multiple cancer‐specific features (e.g., active hTERT promoter AND high miR‐21 AND low miR‐199a), thereby reducing editing in normal cells that may share one but not all of these characteristics. Similarly, Chung et al.^28^ demonstrated a synthetic signalling pathway that senses tumour‐associated antigens and triggers therapeutic effector release, a concept that can be extended to inducible CRISPR‐based interventions.

### 2.7 Comparative summary of inducible platforms

Taken together, the five major inducible CRISPR/Cas architectures differ substantially in leakiness, in vivo dynamic range, delivery burden, and translational readiness (Table S1). Chemically inducible systems are currently the most mature and scalable because they rely on clinically familiar small‐molecule control, but their performance is limited by basal promoter activity, systemic inducer exposure, and pharmacokinetic variability. Light‐inducible systems offer superior spatial precision and rapid activation, but their translational use is restricted by tissue penetration, phototoxicity, and the need for specialized irradiation or upconversion devices. TME‐responsive platforms provide autonomous tumour‐biased activation without external hardware, yet their ON/OFF behaviour depends strongly on heterogeneous hypoxia, pH, redox, enzyme, or miRNA gradients across tumours and patients. Physical‐triggered systems, particularly ultrasound‐, heat‐, and magnetic‐field‐responsive platforms, may be more suitable for deep or locoregional tumours, although uneven energy deposition and device‐dependent safety calibration remain unresolved. Logic‐gated circuits can theoretically achieve the highest specificity by integrating multiple tumour features, but their large payload size, slower circuit kinetics, and complex optimization currently make them less scalable for near‐term clinical translation. Therefore, no single induction class is universally superior; the optimal architecture should be selected according to tumour accessibility, required editing window, acceptable leakiness, delivery route, and manufacturability.

Representative studies illustrate that inducible CRISPR/Cas platforms differ markedly in quantitative ON/OFF performance. The rapamycin‐inducible split‐Cas9 system showed +/− rapamycin activity ratios of 57‐fold, 27‐fold, and 552‐fold at the ASCL1, MYOD1, and IL1RN loci, respectively.^32^ Photoactivatable paCas9 achieved 1.1% indel formation in the dark and 20.5% after blue‐light irradiation at CCR5, corresponding to an approximately 18.6‐fold light‐dependent increase, although its induced activity remained lower than that of full‐length Cas9.^18^ In sgRNA‐level control, theophylline‐responsive aptazyme‐embedded guide RNAs increased GFP disruption from 22% without ligand to 58% with ligand in a reporter assay, whereas endogenous‐locus base editing showed more modest two‐ to threefold ligand dependence under some conditions.^39^ More recently, the multilayer chemically inducible pTET‐DD‐Cas9; AcrIIA4‐LID / pTET: Ultra‐tight circuit achieved a >100‐fold ON/OFF dynamic range for CD81 depletion while maintaining >80% induced editing and near‐background OFF‐state activity.^75^ These examples indicate that inducible control is highly architecture‐, locus‐, and readout‐dependent, and that dynamic range, basal leakiness, and induced editing efficiency should be reported quantitatively rather than inferred from inducibility alone. Because these values were obtained using different cell types, genomic loci, delivery formats, and assays, they should be interpreted as representative benchmarks rather than direct head‐to‐head comparisons.

## 3 APPLICATIONS IN PRECISION ONCOLOGY

**FIGURE 4:**
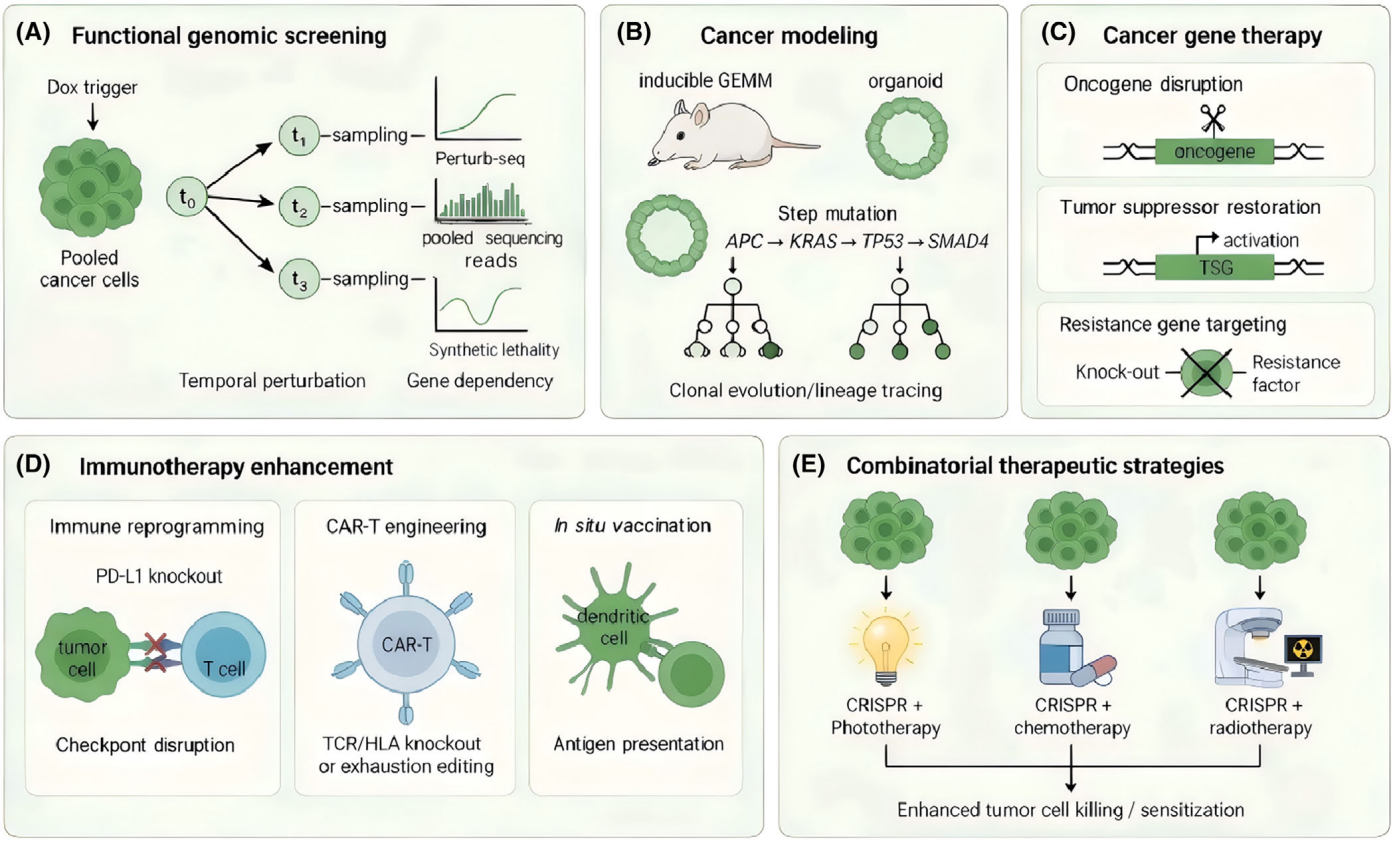
Applications of inducible CRISPR/Cas systems across the precision oncology workflow. Inducible CRISPR/Cas can systems support multiple stages of precision oncology research and therapy. (A) In functional genomic screening, temporally controlled perturbation and sequential sampling enable the identification of gene dependencies and synthetic lethal interactions. (B) In cancer modelling, inducible systems facilitate stepwise mutation introduction in genetically engineered mouse models and organoids, supporting the study of clonal evolution and lineage relationships. (C) In gene therapy, they may support oncogene disruption, tumour suppressor restoration, and resistance gene targeting. (D) In immunotherapy, inducible editing can be used for immune reprogramming, CAR‐T engineering, and in situ vaccination strategies. (E) Inducible CRISPR/Cas can also be integrated with photo‐, chemo‐, and radiotherapy to enhance tumour‐sensitization or therapeutic response. This figure is a conceptual schematic based on the literature cited in the corresponding section and does not contain newly analyzed public datasets.

The integration of inducible CRISPR/Cas systems with precision oncology has created several important applications across cancer research and therapy. This section provides a comprehensive discussion of these applications, organized by functional category (Figure 4).

### 3.1 Functional genomic screening and gene dependency mapping

Genome‐wide CRISPR knockout and activation screens are now widely used to identify cancer vulnerabilities, synthetic lethal interactions, and drug resistance mechanisms.^11^, ^76^, ^77^ However, constitutive CRISPR screens have a fundamental limitation: they capture only the endpoint phenotypic consequences of gene disruption and cannot resolve temporal dynamics of gene function. Inducible CRISPR systems overcome this limitation by enabling time‐resolved perturbation studies.

Dow et al. demonstrated the power of Dox‐inducible Cas9 in performing temporal gene ablation studies in mouse models, revealing that the consequences of tumour suppressor loss depend critically on the developmental stage at which the gene is inactivated.^22^ Similarly, inducible CRISPRi/CRISPRa systems based on Dox‐regulated dCas9‐KRAB (repression) or dCas9‐VPR/VP64 (activation) have been used for reversible gene perturbation screens, enabling the identification of genes whose function is required for tumour maintenance versus tumour initiation—a distinction with profound therapeutic implications.^78^, ^79^

Inducible screens have also been instrumental in dissecting context‐dependent gene essentiality. For example, temporally controlled CRISPR perturbations have been combined with single‐cell RNA sequencing (Perturb‐seq) to map gene regulatory networks at unprecedented resolution,^80^, ^81^ and inducible variants of these approaches have enabled the study of dynamic transcriptional responses over time.

### 3.2 Cancer modelling

Cancer models that capture the genetic complexity and temporal progression of human tumours are essential for preclinical research and drug development. Inducible CRISPR/Cas systems have significantly advanced the field of cancer modelling by enabling the conditional introduction of oncogenic mutations in a tissue‐specific and temporally controlled manner.

#### 3.2.1 Genetically engineered mouse models

Traditional genetically engineered mouse models (GEMMs) based on Cre‐loxP technology require extensive breeding and are limited to pre‐engineered alleles. The advent of constitutive Cas9‐expressing mice (Rosa26‐Cas9) enabled somatic genome editing through viral delivery of sgRNAs, accelerating cancer model generation.^23^ However, inducible Cas9 mice offer further refinements by allowing precise control over the timing and duration of editing. Dow et al. generated Dox‐inducible Cas9 mice (Rosa26‐LSL‐rtTA; Col1a1‐TRE‐Cas9) that enable temporally controlled gene editing in any tissue accessible to Dox.^22^ Using this platform, the authors demonstrated sequential inactivation of tumour suppressors in the intestinal epithelium, faithfully modelling the adenoma‐carcinoma sequence of colorectal cancer.

Platt et al. and Sánchez‐Rivera et al. utilized Cre‐dependent Cas9 mice (Rosa26‐LSL‐Cas9) combined with tissue‐specific Cre drivers (e.g., Krt14‐Cre for skin, Pdx1‐Cre for pancreas) to model tumour initiation in defined compartments.^23^, ^82^ More recently, inducible Cas9 systems have been combined with multiplexed sgRNA libraries delivered via adeno‐associated virus (AAV) to generate autochthonous models harbouring combinatorial mutations in putative cancer genes, enabling in vivo genetic interaction mapping.^83^, ^84^

#### 3.2.2 Organoid models

Three‐dimensional organoid cultures derived from patient tumours or normal tissues have emerged as powerful platforms for cancer modelling and drug testing.^85^ Inducible CRISPR systems have been applied to organoids to model stepwise tumorigenesis ex vivo. Drost et al.^86^ sequentially introduced oncogenic mutations (APC, TP53, KRAS, SMAD4) into normal human intestinal organoids using CRISPR/Cas9, recapitulating the multi‐hit model of colorectal cancer. The use of inducible Cas9 in organoid systems allows researchers to control the timing and order of mutation acquisition, facilitating studies of clonal evolution, epistatic interactions, and the minimal set of driver mutations required for malignant transformation.^87^

#### 3.2.3 Lineage tracing and clonal analysis

Inducible CRISPR‐based lineage tracing systems, such as the CARLIN (CRISPR array repair lineage tracing) mouse, employ Dox‐inducible Cas9 to generate cumulative, heritable barcode mutations in a synthetic target array, enabling retrospective reconstruction of clonal hierarchies and cell fate decisions during tumour evolution.^88^ These systems provide unprecedented insights into cancer stem cell dynamics, clonal competition, and the impact of therapeutic interventions on tumour architecture.

#### 3.2.4 Translationally relevant preclinical models

To improve translational relevance, inducible CRISPR/Cas systems should also be validated in patient‐derived xenografts (PDX), orthotopic tumour models, and humanized immune mouse models. PDX models better preserve patient‐specific tumour heterogeneity and can be used to evaluate tumour penetration, delivery efficiency, and editing responses in clinically relevant tumour architectures. Humanized immune mouse models are particularly important for testing inducible CRISPR strategies designed to enhance immunotherapy, such as checkpoint editing or CAR‐T sensitization. However, establishing stable inducible tumour models remains technically challenging because lentiviral packaging, clonal selection, basal leakiness, and inducer responsiveness may vary across cell lines and in vivo passages.

### 3.3 Cancer gene therapy

Direct therapeutic use of CRISPR/Cas9 in cancer treatment—including oncogene disruption, tumour suppressor restoration, and therapy sensitization—is an active and important area of precision oncology. Inducible systems are particularly valuable in this context, as they are designed to improve tumour‐biased editing while potentially reducing collateral damage to normal tissues.

#### 3.3.1 Oncogene disruption

Inducible CRISPR/Cas systems may enable spatiotemporally controlled oncogene disruption to enhance precision in cancer therapy while potentially reducing off‐target exposure in healthy tissues. For instance, an enzyme‐inducible CRISPR (eiCRISPR) platform achieves cell‐selective genome editing via disease‐associated proteases, restricting Cas9 activity to malignant cells.^89^ Similarly, FUS‐inducible CRISPR systems, delivered via AAVs, disrupt telomeres to sensitize solid tumours to CAR‐T therapy.^90^ Targeting amplified oncogenes (e.g., MYCN in neuroblastoma), CRISPR‐Cas9 nickase exploits replication‐dependent DNA breaks for selective tumour cell killing.^91^

#### 3.3.2 Tumour suppressor restoration

Inducible CRISPR/Cas systems offer precise spatiotemporal control for tumour suppressor gene (TSG) restoration in precision oncology. Catalytically dead Cas9 (dCas9)‐based activators, such as the dCas9‐NVPR complex, effectively restore expression of silenced TSGs (e.g., PER2 and ZNF382), significantly inhibiting malignant phenotypes with high genomic specificity.^92^ Dual CRISPR interference/activation (CRISPRi/a) strategies enable targeted reactivation of non‐mutated X‐linked TSG alleles silenced by X‐chromosome inactivation, presenting a tailored approach for breast cancer therapy.^93^ Furthermore, enhancer‐switching via CRISPR/Cas9‐mediated editing reactivates endogenous TSGs, providing an alternative epigenetic restoration strategy.^94^ Integration of inducible modules—such as enzyme‐responsive (eiCRISPR),^89^ far‐red light‐inducible Cas12a (FICA),^95^ or Tet‐regulated systems^75^—may improve the safety profile by restricting activation to tumour microenvironments(TMEs), potentially reducing off‐target exposure while enabling dynamic control over TSG re‐expression for personalized therapeutic intervention.

#### 3.3.3 Disruption of drug‐resistance genes

Acquired drug resistance is a major cause of treatment failure in oncology. Inducible CRISPR systems can be deployed to disrupt resistance‐conferring genes in a temporally coordinated manner with drug administration. For example, CRISPR‐dCas9‐based platforms enable targeted transcriptional regulation of resistance‐associated genes (e.g., enhancing platinum sensitivity via CT45 modulation) without genomic cleavage, improving drug susceptibility in lung cancer models.^96^ Genome‐wide CRISPR knockout and activation screens systematically identify resistance drivers (e.g., BCL2, BRIP1) and synthetic lethal targets across chemotherapeutics, guiding rational combination strategies.^97^, ^98^, ^99^ Innovations like SIBR‐Cas provide tight, inducible control over editing activity to reduce prolonged nuclease exposure and potentially limit off‐target effects during resistance gene targeting.^100^ Integrating these controllable systems with TME‐responsive delivery holds significant promise for overcoming therapy‐induced resistance while preserving normal tissue function in precision oncology.

### 3.4 Immunotherapy enhancement

The remarkable clinical success of immune checkpoint blockade (ICB) and chimeric antigen receptor (CAR) T‐cell therapy has underscored the critical role of the immune system in cancer control. Inducible CRISPR systems are increasingly being integrated with immunotherapeutic approaches to enhance their efficacy and safety.

#### 3.4.1 Conditional immune checkpoint disruption

Conditional CRISPR/Cas systems may enable more spatiotemporally controlled immune checkpoint disruption to enhance tumour‐specific immunotherapy while potentially reducing systemic toxicity. For example, an HSP70 promoter‐driven CRISPR/Cas9 nanosystem (F‐PC/pHCP) achieves tumour‐selective PD‐L1 genomic knockout, triggering potent T‐cell activation, inhibiting primary and distant tumours, and establishing long‐term immune memory.^101^ Similarly, FUS‐inducible CRISPR platforms allow non‐invasive, localized editing to disrupt checkpoints and prime solid tumours for CAR‐T cell therapy.^90^ Enzyme‐inducible CRISPR (eiCRISPR) further refines specificity by activating editing only in disease‐associated microenvironments via self‐blocked sgRNA unmasking.^89^ These inducible strategies show how controlled immune checkpoint modulation can overcome limitations of conventional blockade therapies.

#### 3.4.2 Inducible CRISPR in CAR‐T cell engineering

Inducible CRISPR has been applied to engineer “armored” CAR‐T cells with enhanced functionality. Examples include: (i) inducible knockout of endogenous TCR and HLA genes to generate universal, allogeneic CAR‐T cells with reduced graft‐versus‐host disease (GvHD) risk^102^; (ii) conditional disruption of T‐cell exhaustion‐associated genes (e.g., TOX, NR4A family) to sustain effector function in the TME^10^; and (iii) inducible activation of synthetic cytokine circuits that augment CAR‐T cell persistence and tumour infiltration.^103^ The temporal control afforded by inducible systems is particularly important for CAR‐T cell engineering, where the timing of gene edits relative to T cell activation and expansion critically influences manufacturing outcomes.

#### 3.4.3 In situ tumour vaccination

In situ tumour vaccination (ISV) leverages endogenous tumour‐associated antigens (TAAs) at the primary tumour site to stimulate systemic, tumour‐specific adaptive immunity while minimizing systemic toxicity.^104^, ^105^ However, its efficacy is frequently constrained by the immunosuppressive TME, insufficient immunogenic cell death (ICD), and inadequate antigen cross‐presentation.^106^, ^107^ Integrating inducible CRISPR/Cas systems offers a precision strategy to overcome these barriers: TME‐responsive nanoplatforms enable programmable release of CRISPR/Cas9 to disrupt immune checkpoints (e.g., PD‐L1, PTPN2) or silence immunosuppressive genes, thereby reprogramming the TME and amplifying adaptive immune responses.^108^ CRISPR activation (CRISPRa) screens further identify targets to enhance antigen presentation and T‐cell infiltration,^106^, ^109^ while manganese‐based or hydrogel‐based delivery systems facilitate localized, stimulus‐triggered genome editing for robust in situ vaccination. These advances position inducible CRISPR/Cas tools may become useful components of next‐generation personalized cancer immunotherapy.

### 3.5 Combinatorial therapeutic strategies

Inducible CRISPR systems are uniquely suited for integration with established cancer treatment modalities, creating synergistic combinatorial strategies.

#### 3.5.1 CRISPR combined with photodynamic/photothermal therapy

Light‐activated CRISPR systems can be co‐delivered with photosensitizers for photodynamic therapy (PDT) or photothermal agents for photothermal therapy (PTT), enabling simultaneous gene editing and physical tumour destruction. Li et al.^110^ developed a multifunctional nanoplatform combining gold nanorods (for PTT), UCNPs (for NIR‐to‐UV light conversion), and photocleavable CRISPR/Cas9 components, achieving synergistic anti‐tumour effects through concurrent gene disruption and hyperthermia. The shared light trigger ensures spatial and temporal coordination of the two therapeutic modalities.

#### 3.5.2 CRISPR combined with chemotherapy

CRISPR/Cas systems synergize with chemotherapy to overcome resistance and enhance tumour‐specific cytotoxicity. Nanoparticle‐mediated co‐delivery of CRISPR components and chemotherapeutics (e.g., cisplatin or doxorubicin) reconfigures cellular drug responses, sensitizes resistant cells via targeted gene knockout (e.g., PRKDC, FBXO44), and targets cancer stem cell‐associated programs while potentially reducing off‐target exposure.^111^, ^112^, ^113^, ^114^ Engineered inducible platforms, such as i‐CRISPR incorporating DNA repair inhibitors, enable spatiotemporally controlled DNA damage to amplify chemotherapy efficacy in a tumour‐selective manner.^115^ These combinatorial strategies reflect a broader move in precision oncology toward individualized and mechanism‐guided treatment combinations.

#### 3.5.3 CRISPR combined with radiotherapy

X‐ray‐guided CRISPR/Cas9 (X‐CC9) enables tumour‐localized activation under irradiation, selectively reprogramming tumour‐associated macrophages (M2‐to‐M1) while sparing healthy tissues.^116^ CRISPR‐based functional screens identify synthetic lethal targets linked to radiation resistance in glioblastoma and head and neck cancers, revealing novel radiosensitization pathways.^117^, ^118^ Knockout of atypical PARP genes (e.g., PARP9/12/13/14) via CRISPR/Cas9 significantly enhances radiosensitivity in colorectal cancer models.^119^ Integrating inducible editing with radiotherapy supports spatiotemporally controlled interventions, advancing personalized combinatorial regimens to overcome therapeutic resistance.^120^ Among current applications, ex vivo immune‐cell engineering and locoregional treatment of accessible solid tumours appear to be more realistic near‐term translational scenarios, whereas systemic in vivo genome editing remains constrained by delivery heterogeneity, tumour penetration, immune responses, and difficulties in monitoring editing outcomes across lesions (Table 2).

**TABLE 2:** Representative applications of inducible CRISPR/Cas systems in oncology.

Application category	Inducible system type	Target gene/pathway	Cancer type	Model	Key finding
Functional genomic screening	Dox‐inducible Cas9/CRISPRi/CRISPRa	Tumour suppressors; temporal dependencies; context‐specific essential genes	Multiple cancers	In vitro + in vivo	Enabled time‐resolved perturbation and helped distinguish genes required for tumour initiation versus maintenance.
Cancer modelling	Dox‐inducible Cas9 mice; Inducible multiplex sgRNA delivery	Apc, Trp53, Kras, Smad4 and other driver combinations	Colorectal, pancreatic and other solid tumours	In vivo GEMM/autochthonous models	Supported sequential introduction of oncogenic lesions and more faithful modelling of tumour evolution.
Cancer modelling	Inducible Cas9 in organoids	APC, TP53, KRAS, SMAD4	Colorectal cancer	Ex vivo organoid	Permitted stepwise mutation engineering and clonal evolution studies in human organoid models.
Lineage tracing	Dox‐inducible barcode editing	Synthetic CRISPR barcode arrays (CARLIN‐like systems)	General oncology/tumour evolution	In vivo	Enabled reconstruction of clonal hierarchies and lineage dynamics during tumour progression.
Gene therapy—oncogene disruption	Enzyme‐inducible CRISPR; FUS‐inducible CRISPR; inducible nickase systems	Telomeres; amplified oncogenic loci; disease‐selective editing programs	Solid tumours; neuroblastoma	In vitro + in vivo	Supported tumour‐biased editing and antitumour effects while potentially reducing collateral editing.
Gene therapy—tumour suppressor restoration	Inducible CRISPRa/far‐red light‐inducible Cas12a/Tet‐regulated circuits	PER2, ZNF382, FOXP3 and other silenced tumour suppressors	Breast cancer and other tumours	In vitro + preclinical	Enabled controlled restoration of endogenous tumour suppressor expression and reduced malignant phenotypes in preclinical models.
Gene therapy—resistance reversal	Inducible CRISPR or CRISPR‐dCas9 regulation combined with drugs	PTEN, CT45, BCL2, BRIP1 and other resistance programs	NSCLC and multiple resistant tumours	In vitro + preclinical	Improved drug sensitivity in preclinical settings and helped identify actionable resistance liabilities.
Immunotherapy enhancement	HSP70/ROS‐responsive or FUS‐inducible checkpoint editing	PD‐L1, immunosuppressive pathways	Solid tumours	In vivo	Tumour‐localized checkpoint disruption promoted T‐cell activation and antitumour responses in preclinical models.
CAR‐T engineering	Inducible CRISPR during ex vivo engineering	TCR/HLA, exhaustion genes, synthetic cytokine modules	Hematologic malignancies and solid tumour CAR‐T settings	Ex vivo/translational	May improve manufacturing flexibility, safety control, persistence, and universality of engineered CAR‐T cells.
In situ vaccination	TME‐responsive CRISPR nanoplatforms; CRISPRa‐guided target discovery	PD‐L1, PTPN2 and TME modulators	Colorectal and other solid tumours	In vivo	Supported TME reprogramming and local‐to‐systemic antitumour immune responses.
Combinatorial therapy	Light‐inducible or nanocarrier‐based inducible CRISPR with PDT/PTT	Gene editing combined with photodynamic/photothermal damage	Solid tumours	In vivo	Shared triggers may coordinate genome editing with local thermal or ROS‐mediated cytotoxicity.
Combinatorial therapy	Inducible CRISPR with chemotherapy or radiotherapy	PRKDC, FBXO44, PARP9/12/13/14, macrophage reprogramming axes	TNBC, glioblastoma, colorectal cancer and others	In vitro + in vivo	Enhanced tumour sensitivity to chemo/radiotherapy in preclinical settings and supported spatiotemporally coordinated combination regimens.

*Note*: The information summarized in this table was extracted from published studies cited in the corresponding section. No independent public dataset re‐analysis was performed.

Abbreviations: CARLIN, CRISPR array repair lineage tracing; CRISPR, clustered regularly interspaced short palindromic repeats; FUS, focused ultrasound; GEMM, genetically engineered mouse model; HLA, human leukocyte antigen; NSCLC, non‐small‐cell lung cancer; PDT, photodynamic therapy; PTT, photothermal therapy; ROS, reactive oxygen species; sgRNA, single‐guide RNA; TCR, T‐cell receptor; TME, tumour microenvironment; TNBC, triple‐negative breast cancer.

### 3.6 Target gene knockout validation in cells and animal models

Target gene knockout in cellular and animal models is essential for verifying the specificity and functional relevance of inducible CRISPR/Cas systems in oncology. Unlike constitutive CRISPR editing, inducible systems should be evaluated in both OFF and ON states to determine basal leakiness, induction efficiency, and target‐dependent phenotypic effects.

In cell‐based models, knockout efficiency can be confirmed by Sanger sequencing, TIDE/ICE analysis, targeted next‐generation sequencing, or amplicon sequencing. Reduced target expression should be further validated at the mRNA and protein levels using qPCR, Western blotting, immunofluorescence, or flow cytometry. Appropriate controls include non‐targeting sgRNA, inducer‐only treatment, untreated cells, and multiple independent sgRNAs targeting the same gene. Rescue experiments with sgRNA‐resistant target gene re‐expression can further confirm that observed phenotypes are specifically caused by target gene disruption.

In animal models, target knockout should be verified directly in tumour tissues or edited organs by targeted sequencing, immunohistochemistry, immunofluorescence, Western blotting, or flow cytometry. Comparison of tumour tissues, adjacent normal tissues, and distant organs is important for evaluating tumour selectivity and potential off‐tumour editing. Functional effects should be linked to downstream pathway changes, tumour growth inhibition, immune activation, drug sensitization, or survival benefit.

**FIGURE 5:**
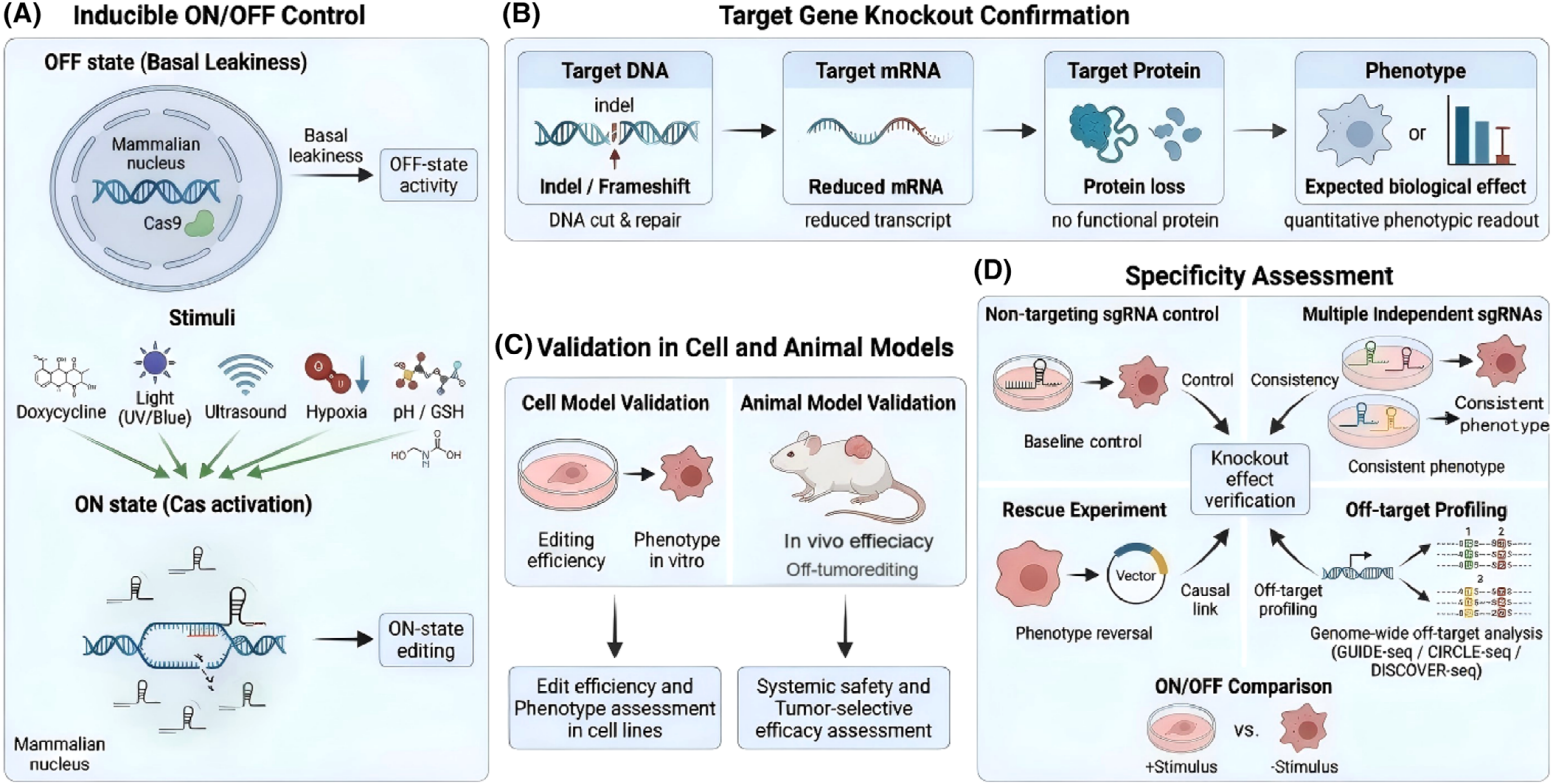
Conceptual framework for validating target gene knockout specificity in inducible CRISPR/Cas systems. Inducible CRISPR/Cas‐mediated knockout should be evaluated in both OFF and ON states to determine basal leakiness, induction efficiency, and dynamic range. Target knockout can be confirmed at the DNA, RNA, protein, and phenotype levels. In cell and animal models, validation should include editing efficiency, phenotypic assessment, tumour‐selective activity, and off‐tumour safety evaluation. Specificity can be further supported by non‐targeting sgRNA controls, multiple independent sgRNAs, rescue experiments, ON/OFF comparison, and off‐target profiling using GUIDE‐seq, CIRCLE‐seq, DISCOVER‐seq, or targeted sequencing of predicted off‐target sites. This figure is a conceptual schematic based on the literature cited in the corresponding section and does not contain newly analyzed public datasets.

Together, validation using multiple sgRNAs, rescue experiments, ON/OFF comparison, and off‐target profiling, such as GUIDE‐seq, CIRCLE‐seq, DISCOVER‐seq, or targeted sequencing of predicted off‐target sites, is critical for demonstrating that inducible CRISPR/Cas systems act through specific target gene knockout in cells and animals. Representative strategies for validating target gene knockout specificity in cellular and animal models are summarized in Figure 5.

## 4 DELIVERY STRATEGIES FOR INDUCIBLE CRISPR/CAS SYSTEMS IN CANCER

**FIGURE 6:**
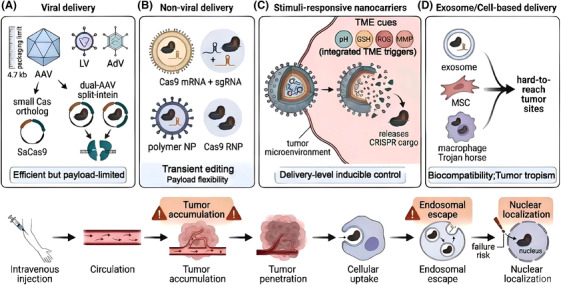
Delivery platforms and in vivo trafficking of inducible CRISPR/Cas systems. Efficient delivery remains a central requirement for the therapeutic translation of inducible CRISPR systems. A, viral vectors, including AAV, lentivirus, and adenovirus, provide high delivery efficiency but are constrained by packaging capacity, payload complexity, and safety considerations. B, non‐viral systems, such as Cas9 mRNA/sgRNA formulations, polymer nanoparticles, and Cas9 ribonucleoproteins, offer transient editing and greater payload flexibility. C, stimuli‐responsive nanocarriers exploit tumour microenvironment triggers, including pH, redox status, reactive oxygen species, and MMPs, to promote conditional CRISPR cargo release CRISPR cargo. (D) Exosome‐ and cell‐based carriers may improve biocompatibility and tumour or inflamed‐tissue tropism. After systemic administration, major biological barriers include circulation, tumour accumulation, tissue penetration, cellular uptake, endosomal escape, and nuclear localization. This figure is a conceptual schematic based on the literature cited in the corresponding section and does not contain newly analyzed public datasets.

The therapeutic translation of inducible CRISPR/Cas systems is critically dependent on the development of efficient, safe, and tumour‐targeted delivery vehicles. The added complexity of inducible systems—which often involve multiple components (Cas9, sgRNA, inducer, regulatory elements)—places additional demands on delivery platforms (Figure 6).

### 4.1 Viral delivery

Adeno‐associated virus (AAV), lentivirus (LV), and adenovirus (AdV) remain the most widely used vectors for in vivo delivery of CRISPR components. AAV is particularly favoured for its low immunogenicity, broad tissue tropism, and established clinical safety profile.^121^ However, the limited packaging capacity of AAV (∼4.7 kb) poses challenges for accommodating the large SpCas9 gene (∼4.2 kb) together with inducible regulatory elements. Strategies to address this limitation include: (i) the use of smaller Cas orthologs, such as Staphylococcus aureus Cas9 (SaCas9, ∼3.2 kb) or CjCas9 (∼2.9 kb)^122^, ^123^; (ii) dual‐AAV split‐intein systems that deliver the two halves of Cas9 in separate vectors^34^; and (iii) the use of the smaller Cas12a (Cpf1) nucleases.^124^ For inducible systems, the regulatory cassette (e.g., rtTA‐TRE for Dox induction) adds ∼1–2 kb to the payload, further necessitating compact designs or multi‐vector approaches.

### 4.2 Non‐viral delivery

Non‐viral delivery systems offer several advantages over viral vectors, including reduced immunogenicity, absence of insertional mutagenesis risk, and greater payload flexibility. LNPs have emerged as the leading non‐viral platform for CRISPR delivery, buoyed by the clinical success of LNP‐formulated mRNA vaccines.^125^ LNPs can efficiently encapsulate Cas9 mRNA and sgRNA or preformed Cas9‐sgRNA ribonucleoprotein (RNP) complexes, enabling transient editing with reduced off‐target risk compared to DNA‐based delivery.^126^

Stimulus‐responsive nanocarriers add an additional layer of spatial and temporal control by releasing their CRISPR payload only in response to specific triggers within the TME. Examples include: (i) pH‐responsive polymeric nanoparticles that disassemble in the acidic extracellular milieu of tumours^58^; (ii) GSH‐responsive disulfide‐crosslinked nanocapsules that release Cas9 RNPs in the reducing intracellular environment^127^; (iii) ROS‐responsive nanocarriers that exploit the elevated oxidative stress in tumours^29^; and (iv) enzyme‐responsive systems that are degraded by tumour‐associated matrix metalloproteinases (MMPs), releasing CRISPR components at the tumour site.^128^

### 4.3 Exosome and cell‐based delivery

Exosomes and extracellular vesicles (EVs) offer a bio‐inspired delivery platform with intrinsic biocompatibility and the ability to cross biological barriers, including the blood–brain barrier. Kim et al.^123^ demonstrated that Cas9‐loaded exosomes (termed “exosome‐mediated CRISPR/Cas9 delivery” or ExoCRISPR) could achieve efficient gene editing in target cells with minimal immunogenicity. Furthermore, cell‐based delivery strategies using tumour‐tropic mesenchymal stem cells (MSCs) or macrophages as “Trojan horse” carriers of inducible CRISPR systems have been explored for targeted gene editing in hard‐to‐reach tumour sites (Table 3).^129^

**TABLE 3:** Comparison of delivery vehicles for inducible CRISPR/Cas systems in cancer.

Vector type	Packaging capacity	Immunogenicity	Insertional mutagenesis risk	Tumour‐targeting ability	Clinical translation stage	Compatibility with inducible systems
AAV	∼4.7 kb per vector; often requires smaller Cas proteins or dual‐AAV designs	Low to moderate; pre‐existing anti‐capsid immunity remains relevant	Very low	Good after capsid selection, local injection or tissue‐tropic serotypes; systemic solid‐tumour targeting still challenging	Clinically advanced viral vector for genome editing	Compatible with compact inducible modules; large multi‐component circuits are difficult unless split across vectors.
Lentivirus (LV)	∼8–10 kb	Moderate	Present because of genomic integration	Useful ex vivo and for dividing cells; less ideal for in vivo solid‐tumour precision delivery	Clinically established mainly for ex vivo cell engineering	Good payload space for Tet or logic circuits, but persistent integration is a safety concern for therapeutic editing.
Adenovirus (AdV)	∼8 kb for first‐generation; much larger for helper‐dependent systems	Moderate to high	Negligible integration risk	High transduction efficiency; can be retargeted but liver/immunity issues remain	Clinically used in gene therapy/oncolytic virotherapy contexts	Strong for large inducible cassettes, but innate immunity and transient expression must be managed.
Lipid nanoparticles (LNP)	Flexible for Cas9 mRNA + sgRNA or RNP cargo; not limited by viral genome rules	Low to moderate and formulation‐dependent	None	Improving with ligand decoration and organ‐selective formulation; tumour accumulation still heterogeneous	Rapidly advancing non‐viral clinical platform	Highly compatible with transient inducible editing and stimulus‐responsive release strategies; strong fit for mRNA/RNP‐based systems.
Polymeric nanoparticles	Flexible; can load DNA, mRNA or RNP	Usually low to moderate	None	Can be engineered for pH/GSH/ROS/MMP responsiveness and active targeting	Mostly preclinical	Compatible with TME‐responsive and externally triggered release, though batch reproducibility can be limiting.
Exosomes / EVs	Flexible but variable; typically suited for RNP, RNA and small nucleic acid payloads	Low	None	Intrinsic biocompatibility and some barrier‐crossing advantages; targeting depends on source engineering	Early preclinical to translational exploration	Compatible with transient and low‐immunogenicity inducible systems, but loading efficiency and standardization remain challenging.
Cell carriers (MSC, macrophage, T cell, other tumour‐tropic cells)	Effectively high because cargo can be produced intracellularly or loaded ex vivo	Carrier‐dependent; can be favourable autologously	Depends on whether cargo is integrated into carrier cells	Potential tumour or inflamed‐tissue tropism	Mostly preclinical/translational	Attractive for complex multi‐component inducible systems, especially when homing or local release is required, but manufacturing is complex.

*Note*: The information summarized in this table was extracted from published studies cited in the corresponding section. No independent public dataset re‐analysis was performed.

Abbreviations: AAV, adeno‐associated virus; AdV, adenovirus; EVs, extracellular vesicles; GSH, glutathione; LNP, lipid nanoparticle; LV, lentivirus; MMP, matrix metalloproteinase; MSC, mesenchymal stem cell; RNP, ribonucleoprotein; ROS, reactive oxygen species; sgRNA, single‐guide RNA; TME, tumour microenvironment.

## 5 CHALLENGES AND LIMITATIONS

Despite the remarkable progress outlined above, several significant challenges must be addressed before inducible CRISPR/Cas systems can be translated into clinical oncology applications.

### 5.1 Leaky expression and background activity

A persistent concern with inducible systems is residual (“leaky”) activity in the uninduced state, which can lead to unintended editing and compromise the safety of therapeutic applications. Tet‐inducible systems, in particular, are notorious for low‐level basal transcription from the TRE promoter even in the absence of Dox.^130^ Strategies to minimize leakiness include the use of tighter repressor systems (e.g., incorporating the KRAB silencing domain), insulator elements, and dual‐input AND‐gate architectures that require two independent signals for activation.^131^

### 5.2 Induction efficiency and dynamic range

Inducible CRISPR/Cas systems face critical hurdles in oncology applications, particularly regarding induction efficiency and dynamic range. Achieving low basal activity with robust, tunable activation remains challenging; systems like Branaplam‐regulated splice switches or Tet‐inducible CRISPRi aim for tighter transcriptional control but often exhibit variable responsiveness across TMEs.^75^ Chemical or enzyme‐inducible designs (e.g., eiCRISPR) struggle with inconsistent trigger penetration and delayed kinetics in restrictive tumour niches, limiting precise spatiotemporal editing.^132^ Additionally, split or allosteric architectures may suffer from incomplete reconstitution or narrow activation windows, compromising therapeutic specificity in heterogeneous cancer contexts.^100^, ^133^

### 5.3 Tissue penetration of physical stimuli

A critical limitation of physical stimuli‐responsive CRISPR/Cas systems in oncology lies in the restricted tissue penetration depth of certain triggers. Light‐based activation, for instance, suffers from shallow penetration in dense tumour tissues due to scattering and absorption, hindering precise genome editing in deep‐seated malignancies.^95^ Although ultrasound and magnetic stimuli exhibit improved tissue penetration capabilities, achieving uniform stimulus distribution and sufficient energy delivery across heterogeneous TMEs remains challenging.^74^ These physical barriers compromise spatiotemporal control fidelity and editing efficiency at target sites, particularly in solid tumours with complex stromal architecture.^134^ Advancing stimulus‐delivery technologies—such as optimizing wavelength parameters or integrating multimodal activation strategies—is essential to overcome penetration constraints and unlock the full therapeutic potential of physically inducible CRISPR platforms in precision oncology.

### 5.4 Immunogenicity

Cas proteins are foreign antigens that can elicit both humoral and cellular immune responses. Pre‐existing immunity to SpCas9 and SaCas9 has been detected in a significant proportion of the human population due to prior Streptococcus exposure.^14^, ^15^ While inducible systems that limit the duration and level of Cas protein expression may partially mitigate this concern, the immunogenicity of the inducible regulatory components themselves (e.g., bacterial rtTA, plant‐derived photoreceptors) must also be considered.^135^

### 5.5 Delivery challenges

Because inducible CRISPR systems often contain multiple components, they place substantial demands on delivery vehicles. Co‐packaging Cas9 protein/mRNA, sgRNA, and regulatory elements (e.g., rtTA, split dimerization domains) within a single nanoparticle is technically challenging, and the stoichiometric optimization of each component is critical for maximal inducibility.^136^ Moreover, all components must reach the tumour, enter target cells, escape endosomes, and localize to the nucleus, which remains a major bioengineering challenge.^137^

### 5.6 Off‐target editing

Although inducible systems can reduce off‐target effects by limiting the duration of Cas9 activity, they do not eliminate them entirely. The combination of inducible strategies with high‐fidelity Cas9 variants (e.g., eSpCas9, SpCas9‐HF1, HypaCas9, evoCas9) offers a synergistic approach to minimize off‐target mutagenesis.^138^, ^139^, ^140^ Comprehensive off‐target profiling using unbiased methods such as GUIDE‐seq, CIRCLE‐seq, and DISCOVER‐seq will be essential for the clinical development of inducible CRISPR therapeutics.^141^, ^142^

## 6 FUTURE PERSPECTIVES

### 6.1 Next‐generation inducible architectures

The continued expansion of the CRISPR toolkit—including Cas12, Cas13, CasX, CasΦ, and newly discovered miniature Cas variants—provides a growing repertoire of effectors amenable to inducible engineering.^143^, ^144^ The smaller size of these proteins facilitates packaging within AAV vectors and reduces the immunogenic footprint. Furthermore, the development of multi‐orthogonal inducible systems (e.g., combining Dox‐inducible Cas9 with light‐inducible Cas12a) will enable multiplexed, independently controlled editing of multiple genomic targets, a capability of particular value for disrupting the polygenic networks that drive cancer.^145^

### 6.2 Closed‐loop “sense‐and‐respond” systems

Future developments will focus on engineering CRISPR‐based closed‐loop sense‐and‐respond circuits that dynamically detect tumour‐specific biomarkers (e.g., miRNAs, enzymes like APE1) and trigger precise therapeutic outputs without external intervention. Integrating incoherent feedforward loop (iFFL) architectures with inducible Cas13/Cas12 effectors can stabilize gene expression amid cellular noise while enabling tunable adaptation responses for multiplexed control.^146^, ^147^ Modular systems such as CRISPR‐ADAReader, featuring programmable positive/negative feedback loops, allow cell‐type‐specific monitoring and reprogramming of oncogenic pathways in response to endogenous RNA cues.^148^, ^149^ Coupling these circuits with stimuli‐responsive delivery carriers (e.g., TME‐activated nanoparticles) may further enhance spatiotemporal precision in solid tumours.^132^, ^150^

### 6.3 Epigenome editing for cancer epigenetics

Beyond genetic editing, inducible CRISPR‐based epigenome editors (e.g., dCas9 fused to DNMT3A, TET1, p300, or LSD1) offer the potential for reversible modulation of the cancer epigenome. Given that many cancer‐associated epigenetic alterations are potentially reversible—unlike genetic mutations—inducible epigenome editing provides a uniquely powerful tool for dissecting the causal role of specific epigenetic marks in tumorigenesis and for developing epigenetic therapies.^151^, ^152^ The reversibility of both the inducible control and the epigenetic modification itself makes this approach particularly attractive for therapeutic applications where permanent genome alteration is undesirable.

### 6.4 RNA‐level editing for cancer transcriptomics

Inducible Cas13‐based RNA editors enable programmable RNA knockdown or RNA base editing without permanent alteration of the genome, offering a transient and reversible modality for cancer therapy.^9^, ^153^ The development of light‐inducible and chemically inducible Cas13 systems has been reported, enabling temporal control over RNA‐level interventions.^153^, ^154^ These RNA‐targeting approaches may be particularly suitable for degrading oncogenic fusion transcripts, blocking splice variants associated with drug resistance, or modulating non‐coding RNA networks that regulate tumour progression.

### 6.5 Clinical translation and regulatory considerations

At present, inducible CRISPR/Cas systems for oncology remain largely preclinical, whereas most clinically tested CRISPR‐based cancer therapies have used ex vivo editing of immune cells, such as PD‐1‐edited T cells for advanced non‐small‐cell lung cancer and CRISPR‐edited tumour‐infiltrating lymphocytes.^155^, ^156^ These studies suggest that ex vivo immune‐cell engineering may be the nearest‐term translational route because edited cells can be expanded, characterized, and quality‐controlled before reinfusion.^155^, ^156^ By contrast, systemic in vivo tumour delivery remains constrained by heterogeneous tumour accumulation, limited tissue penetration, immune responses, and difficulty in monitoring editing outcomes across lesions.^157^

Clinical‐grade manufacturing of inducible CRISPR/Cas systems presents additional challenges because these platforms often require coordinated production of Cas proteins or mRNA, sgRNAs, regulatory modules, delivery vehicles, and sometimes external inducers. Under good manufacturing practice (GMP) conditions, critical quality attributes should include component purity, vector or nanoparticle size distribution, encapsulation efficiency, editing potency, ON/OFF dynamic range, basal leakiness, off‐target profile, sterility, endotoxin level, and batch‐to‐batch consistency. For multi‐component systems, the stoichiometric ratio among Cas, sgRNA, and regulatory elements must also be controlled. These manufacturing and quality‐control requirements may strongly influence which inducible architectures are realistic candidates for translation.

Future clinical translation of inducible CRISPR/Cas systems will require better delivery precision, careful control of off‐target risks and immunogenicity, and further development of TME‐responsive platforms.^132^, ^158^ Regulatory pathways must evaluate temporal control mechanisms—such as enzyme‐inducible (eiCRISPR) or chemically triggered systems—to ensure cell‐selective editing safety and minimize genotoxicity.^89^, ^159^ Standardizing preclinical validation of inducible components (e.g., split‐crRNA designs for multi‐target discrimination) and harmonizing biomarker‐driven trial frameworks will be critical for regulatory approval.^160^ At the same time, non‐viral delivery vectors tailored to inducible architectures must be optimized to overcome physiological barriers and meet biosafety requirements.^161^

In addition to immunogenicity and off‐target editing, virus‐delivered, multi‐component CRISPR/Cas systems raise broader biosafety, environmental, and ethical concerns. Potential risks include long‐term vector persistence, non‐target tissue distribution, vector shedding, unintended recombination, and imbalanced expression of Cas proteins, sgRNAs, and regulatory modules. Although environmental risks are likely limited in oncology settings, they should be assessed for replication‐competent or shedding‐prone vectors. Ethically, patients should be informed about the irreversible nature of genome editing, the uncertain long‐term effects, and the need for extended follow‐up.

For surgically accessible solid tumours, inducible CRISPR/Cas systems may be particularly attractive as locoregional or perioperative interventions rather than as first‐line systemic therapies. Image‐guided intratumoral injection, intraoperative exposure of the tumour bed, or postoperative hydrogel‐based local release could reduce systemic biodistribution and facilitate safety monitoring. In thyroid cancer and other resectable solid tumours, such strategies could be explored in neoadjuvant or adjuvant settings to sensitize residual tumour cells to radiotherapy, immunotherapy, or targeted therapy while limiting editing activity in normal tissues. This clinical scenario may provide a more realistic translational path than systemic in vivo genome editing, especially for platforms activated by ultrasound, heat, light, or tumour microenvironmental cues.

### 6.6 Artificial intelligence‐guided design

Machine learning (ML) and artificial intelligence (AI) are increasingly being applied to CRISPR technology and may help improve inducible system design. AI‐guided approaches can be used to: (i) predict optimal sgRNA sequences and off‐target risks using deep learning‐based guide design models^162^, ^163^; (ii) design inducible regulatory elements with desired dynamic properties using deep learning models trained on high‐throughput functional data^163^; (iii) optimize nanoparticle formulations for stimulus‐responsive delivery^164^; (iv) identify cancer‐specific promoter‐miRNA combinations for multi‐input logic gates through integrative analysis of cancer genomic and transcriptomic databases^165^; and (v) assist Cas protein engineering through structural prediction and protein‐design frameworks, such as AlphaFold‐like protein modelling tools. However, current AI/ML‐guided design still faces several practical limitations, including a lack of high‐quality standardized training datasets, weak generalizability across cell types and from in vitro systems to in vivo tumour contexts, and limited interpretability of model predictions.

## 7 CONCLUSIONS AND FUTURE ROADMAP

Inducible CRISPR/Cas systems represent an important step in genome editing because they add temporal and spatial control to a technology that is otherwise highly active but difficult to restrict to diseased tissue. By placing genome‐editing activity under the governance of chemical, optical, endogenous biological, or physical stimuli, these systems are designed to address translational bottlenecks that have long hindered CRISPR‐based cancer therapies: off‐target genotoxicity, immunogenicity, and the inability to achieve tumour‐selective editing.

Although increasingly sophisticated inducible architectures have been developed, ranging from transcriptional switches to multi‐input logic gates, the field still faces several translational barriers. The gap between proof‐of‐concept demonstrations in cell culture and clinically viable cancer therapeutics remains substantial. To support clinical translation, three issues deserve particular attention.

### 7.1 Three critical milestones for the next five years

#### 7.1.1 Milestone 1: Establish quantitative benchmarks for in vivo performance

The field currently lacks standardized metrics to compare inducible systems across platforms. Consensus is needed on:

‐ Minimum acceptable dynamic range (fold‐change between ON/OFF states) in solid tumours, not just cell culture
‐ Maximum tolerable leakiness (uninduced activity as % of maximal activity) for different therapeutic contexts
‐ Editing efficiency thresholds required for phenotypic impact in heterogeneous tumour populations
‐ Standardized tumour models for head‐to‐head comparisons (e.g., orthotopic vs. subcutaneous, immunocompetent vs. immunodeficient)

Without these benchmarks, we cannot objectively assess whether a “tighter” system with 10% editing efficiency is superior to a “leakier” system with 60% efficiency. The community should establish a consortium to define these parameters through systematic comparative studies, similar to what the ENCODE project achieved for functional genomics.

#### 7.1.2 Milestone 2: Solve the delivery‐inducibility trade‐off

Current inducible systems face a paradox: the more components required for tight control (split proteins, regulatory RNAs, stabilizing ligands), the harder they become to deliver efficiently. We must prioritize:

‐ All‐in‐one compact architectures: Engineering miniature Cas variants (CasΦ, Cas12f) with built‐in inducibility through rational protein design or directed evolution, eliminating the need for separate regulatory modules.
‐ Stimulus‐responsive delivery vehicles that ARE the inducible switch: Rather than delivering pre‐assembled inducible systems, develop nanocarriers where the act of cargo release itself constitutes the induction event (e.g., TME‐triggered disassembly releases active Cas9 RNPs).
‐ Tissue‐specific AAV capsids with endogenous promoters: Combine viral tropism engineering with tumour‐specific transcriptional control to achieve dual‐layer selectivity without payload expansion.

The goal is not merely incremental improvement but a fundamental redesign: inducible systems must become simpler to deliver, not more complex.

#### 7.1.3 Milestone 3: Demonstrate durable safety in large animal models

Before any inducible CRISPR cancer therapy enters human trials, we need long‐term (≥6 month) safety data in immunocompetent large animals (pigs, non‐human primates) addressing:

‐ Chronic immunogenicity: Do repeated inductions trigger adaptive immune responses that eliminate edited cells or cause systemic inflammation?
‐ Off‐target accumulation: Does transient Cas9 activity truly reduce off‐target editing, or do repeated induction cycles lead to cumulative mutagenic burden?
‐ Resistance evolution: Can tumours evolve to evade inducible systems (e.g., by silencing tumour‐specific promoters or upregulating anti‐CRISPR‐like inhibitors)?
‐ Biodistribution of inducers: For systemically administered chemical or physical triggers, what are the long‐term consequences of repeated exposure?

These studies are expensive and time‐consuming, but they are non‐negotiable. The field cannot afford a clinical failure due to unforeseen toxicity that could have been detected in appropriate preclinical models.

### 7.2 A call for disciplined innovation

The allure of engineering ever‐more elaborate inducible circuits—triple‐input AND gates, nested feedback loops, orthogonal multi‐effector systems—is undeniable. But we must resist the temptation to prioritize complexity over clinical viability. The most successful inducible system will not be the most intellectually elegant; it will be the one that is:

1. Simple enough to manufacture at clinical scale
2. Robust enough to function in the chaotic TME
3. Safe enough to gain regulatory approval
4. Effective enough to improve patient outcomes

This requires a cultural shift: from publishing incremental variations on existing themes to tackling the hard problems of delivery, immunogenicity, and long‐term safety. It requires collaboration between genome engineers, cancer biologists, immunologists, materials scientists, regulatory experts, and—critically—patients and clinicians who understand what endpoints truly matter.

### 7.3 The path forward

If the field can achieve these three milestones, the next decade will witness the emergence of “smart” CRISPR therapeutics that autonomously sense tumour‐specific signals, execute precise genomic interventions, and then self‐inactivate—all without external intervention. These systems will not replace conventional cancer therapies but will synergize with them: sensitizing resistant tumours to chemotherapy, reprogramming immunosuppressive microenvironments to enhance checkpoint blockade, and selectively eliminating cancer stem cells that drive relapse.

The convergence of next‐generation Cas effectors, closed‐loop synthetic circuits, AI‐guided design, and stimulus‐responsive nanomaterials creates an important opportunity for the field. But opportunity alone does not translate to clinical impact. That requires disciplined focus on the translational barriers that separate proof‐of‐concept from proof‐of‐efficacy.

### 7.4 Author's perspective: Most promising near‐term architectures

In our view, two inducible CRISPR/Cas architectures are most likely to advance toward clinical oncology within the next five years. The first is ex vivo immune‐cell engineering using transient or chemically controllable CRISPR modules, because edited cells can be expanded, quality‐controlled, screened for off‐target events, and functionally validated before reinfusion. This route aligns better with current clinical experience in CRISPR‐edited T cells than systemic in vivo tumour editing. The second is locoregional stimulus‐responsive delivery of Cas mRNA or RNPs for surgically accessible solid tumours, particularly when ultrasound, heat, light, or TME‐responsive nanocarriers can restrict editing to the tumour bed or residual disease. By contrast, highly complex multi‐input logic‐gated systems are scientifically attractive but are less likely to reach clinical testing soon because of payload size, manufacturing complexity, and delivery uncertainty.

Existing preclinical studies indicate that inducible CRISPR systems can function in cancer models. The next challenge is to determine whether these systems can be optimized, validated, and delivered safely enough to benefit patients. Progress over the next several years will depend on rigorous preclinical testing, clinically realistic delivery strategies, and careful evaluation of long‐term safety.

## AUTHOR CONTRIBUTIONS

**Ziliang Ding and Yukun Wei**: Conceptualization, methodology, investigation, formal analysis, writing—original draft; **Yong Han and Pengfei Gu**: Conceptualization, supervision, writing—review and editing, project administration. All the authors have approved the final article for publication and are accountable for this work.

## AUTHOR DISCLOSURE STATEMENT

The authors have no relevant disclosures to report.

## DECLARATION OF AI‐ASSISTED FIGURE PREPARATION

Artificial intelligence‐assisted tools, specifically Nano Banana, were used only to assist with preliminary schematic figure generation and visual refinement. The figures were subsequently revised using WPS Office. No artificial intelligence tools were used to generate the manuscript text, scientific content, data interpretation, or conclusions. All figure labels, terminology, conceptual organization, scientific content, and final figure validation were independently reviewed, edited, and approved by the authors.

## CONFLICT OF INTEREST STATEMENT

The authors declare no conflict of interest.

## ETHICS STATEMENT

This review article does not involve any original research with human participants, human data, or animal subjects conducted by the authors. All content is based on previously published literature.

## Supporting information



Supporting Information

## Data Availability

No new data were generated or analyzed in this review. All information is derived from publicly available references cited in the manuscript.
