# Supplementary material for: Inducible CRISPR/Cas systems in precision oncology: Current applications and future perspectives
Source: Clin Transl Med. 2026 Jun 15;16(6):e70720. doi: 10.1002/ctm2.70720 (PMC13269835; doi:10.1002/ctm2.70720)
Supplement: Supplementary file 1 — Supporting Information [file CTM2-16-e70720-s001.docx]

**Table S1. Comparative guidance for major inducible CRISPR/Cas platforms**

| **Induction type** | **Representative systems** | **ON/OFF control / leakiness** | **Temporal resolution / reversibility** | **In vivo compatibility** | **Scalability / translational feasibility** | **Refs.** |
| --- | --- | --- | --- | --- | --- | --- |
| Chemical | Tet-On/Tet-Off, split-Cas9 CID, inteins, destabilizing domains, ligand-responsive sgRNAs | Moderate to high; leakiness depends on promoter, dimerizer, or destabilizing module | Hours to days;  often reversible after inducer withdrawal | Moderate;  limited by inducer pharmacokinetics and systemic exposure | Relatively scalable;  clinically familiar small-molecule control | [17, 22, 32] |
| Light | paCas9, split photoactivatable Cas9, light-responsive promoters, caged sgRNAs | High spatial control; low basal activity in well-optimized systems | Seconds to hours; reversibility depends on the photoswitch design | Limited for deep tumors unless combined with NIR/UCNP strategies | Moderate;  requires light-delivery hardware | [18, 42, 47] |
| TME-responsive | Hypoxia-, pH-, GSH-, ROS-, enzyme-, or miRNA-responsive systems | Context-dependent; leakiness depends on tumor-normal contrast of the cue | Usually slower and less externally tunable; reversibility depends on cue dynamics | Potentially high for solid tumors with strong TME gradients | Moderate;  compatible with nanocarriers and synthetic circuits | [51, 56, 59] |
| Physical | Ultrasound, magnetic, thermal, NIR/UCNP-triggered systems | Moderate to high when the stimulus is spatially focused | Minutes to hours; externally tunable and often repeatable | Moderate to high; ultrasound, magnetic, and NIR approaches improve tissue access | Moderate;  depends on device availability and safety calibration | [69, 70, 75] |
| Logic-gated | AND/OR/NOT promoter-miRNA gates, multi-input synthetic circuits | Potentially high specificity;  lower leakiness if inputs are well selected | Variable;  depends on transcriptional or RNA-circuit kinetics | Currently limited, mostly preclinical | Lower scalability due to circuit complexity and payload burden | [27, 28] |

**Abbreviations:** Acr, anti-CRISPR protein; CID, chemically induced dimerization; CRISPR/Cas, clustered regularly interspaced short palindromic repeats/CRISPR-associated protein; FUS, focused ultrasound; GSH, glutathione; HIF, hypoxia-inducible factor; miRNA, microRNA; NIR, near-infrared; ROS, reactive oxygen species; sgRNA, single-guide RNA; TME, tumor microenvironment; UCNP, upconversion nanoparticle. The information summarized in this table was extracted from published studies cited in the corresponding section. No independent public dataset re-analysis was performed.
